# Molecular mechanisms of insulin resistance and altered carbohydrate metabolism in PCOS: a scoping review

**DOI:** 10.3389/fendo.2026.1810805

**Published:** 2026-04-13

**Authors:** Mikołaj Kisiała, Wiktoria Mazepa, Dominika Bauer, Patrycja Tabaka, Michał Gas, Aleksander Sowiński, Krzysztof Wolak, Albert Synal, Julia Gąsiorowska, Oliwia Sas, Wojciech Urbański, Oliwier Pioterek, Mateusz Mazurek, Zygmunt Domagała

**Affiliations:** 1 Division of Anatomy, Department of Human Morphology and Embryology, Wroclaw Medical University, Wroclaw, Poland; 2 1st Department of Obstetrics and Gynecology, Medical University of Warsaw, Warsaw, Poland; 3 Clinical and Dissecting Anatomy Scientific Club, Wroclaw Medical University, Wroclaw, Poland; 4 Faculty of Medicine, Wroclaw Medical University, Wroclaw, Poland

**Keywords:** carbohydrate metabolism, energy metabolism, insulin resistance, mitochondrial dysfunction, PI3K-AKT signaling, polycystic ovary syndrome

## Abstract

Polycystic ovary syndrome (PCOS) is a common endocrinopathy affecting women of reproductive age, characterized by oligo- or anovulation, hyperandrogenism, and polycystic ovarian morphology. Beyond its reproductive manifestations, PCOS is increasingly recognized as a complex endocrine–metabolic disorder frequently associated with impaired carbohydrate metabolism and insulin resistance, often independent of body mass. Despite extensive research, the molecular mechanisms underlying insulin resistance across metabolic and reproductive tissues in PCOS remain incompletely characterized. This scoping review aimed to systematically map molecular disturbances in insulin signaling and carbohydrate metabolism in PCOS, explore associations between tissue-specific mechanisms, and identify key gaps in the current evidence. We included peer-reviewed original studies published in English between January 2018 and May 2025, retrieved from PubMed, Embase, and Web of Science, that investigated molecular or cellular pathways related to insulin resistance or glucose metabolism in PCOS. The available evidence predominantly addressed granulosa cells and ovarian tissue, with additional data from endometrium, liver, adipose tissue, skeletal muscle, pancreatic beta-cells, and systemic regulatory pathways. Recurrent mechanisms underlying insulin resistance in PCOS included post-receptor defects in IRS/PI3K/AKT and MAPK signaling, impaired GLUT4 expression and trafficking, mitochondrial and glycolytic dysfunction, chronic low-grade inflammation, androgen receptor–mediated metabolic reprogramming, circadian rhythm disruption, and epigenetic or environmental modulators. Evidence from human studies remains limited, with many proposed molecular mechanisms being supported predominantly by rodent or cell line models. To translate this knowledge to clinical and therapeutic application, additional high-quality longitudinal human research with comprehensive multi-omics is necessary to validate key mechanisms in ovarian and metabolic tissues, especially those involving IRS/PI3K/AKT signaling, GLUT4 regulation, inflammation, and androgen-driven metabolic dysfunction.

## 1 Introduction

Polycystic ovary syndrome (PCOS) is one of the most prevalent endocrinopathies affecting 6-13% of women of reproductive age worldwide (1). Beyond its reproductive manifestations, PCOS is increasingly recognized as a complex endocrine–metabolic disorder. Dysregulation of carbohydrate metabolism is a key feature of PCOS, yet its underlying mechanisms have not been fully elucidated. Insulin resistance (IR) is present in 75–95% of women with PCOS, independently of body mass index (2).

Consequently, impaired glucose homeostasis substantially increases the risk of type 2 diabetes mellitus, cardiovascular disease, and reduced fertility, contributing to the long-term cardiometabolic burden associated with PCOS. The most recent international evidence-based guidelines for PCOS emphasize the importance of early diagnosis and individualized management of metabolic complications in affected women (3).

However, while the clinical relevance of glucose dysregulation in PCOS is well established, the molecular mechanisms linking endocrine disturbances to impaired insulin signaling and carbohydrate metabolism remain incompletely understood.

To date, molecular mechanisms underlying carbohydrate metabolism dysregulation in PCOS have been investigated only fragmentarily, with much of the available evidence derived from animal models or *in vitro* cell systems. Although these studies have provided important mechanistic insights, the literature remains highly heterogeneous in the tissues examined, experimental models, and molecular pathways assessed.

Given the breadth, heterogeneity, and interdisciplinary nature of the available literature, a scoping review methodology is particularly well-suited to this topic. This approach allows comprehensive mapping of the extent, nature, and types of evidence without formal risk-of-bias assessment, which would be challenging and potentially inappropriate for mechanistic molecular studies.

This scoping review aims to map and categorize the molecular mechanisms underlying dysregulation of insulin signaling and carbohydrate metabolism in PCOS, and to identify key gaps that warrant further investigation.

Following publication of the 2018 International Evidence-Based Guideline for PCOS (4), PCOS was recognized as an insulin-resistant metabolic disorder. At the same time, the guideline noted that available tests for IR lacked sufficient accuracy for diagnostic use and therefore should not be incorporated into the diagnostic criteria. Instead, it recommended clinical monitoring of glycemic status, including baseline assessment and repeated evaluation every 1–3 years, with oral glucose tolerance testing reserved for high-risk individuals and offered preconception to those planning pregnancy or seeking fertility treatment. Together, these recommendations underscore the clinical importance of glucose dysregulation in PCOS and justify a focused scoping review of studies published from 2018 onward that examine the molecular mechanisms of IR and altered carbohydrate metabolism.

The review questions were structured according to the Joanna Briggs Institute (PCC - Population - Concept - Context) model (5):

Population (P): human or animal studies, as well as *in vitro* or *in vivo* experimental models, analyzing disturbances in insulin tolerance or carbohydrate metabolism in PCOS models or in women with PCOS.

Concept (C): alterations in molecular pathways, receptors, mitochondrial function, or epigenetic regulation that contribute to IR or altered carbohydrate metabolism in PCOS.

Context (C): any research setting, with no geographical restrictions; studies published in English.

## 2 Materials and methods

### 2.1 Reporting guidelines

This scoping review was conducted in accordance with the Joanna Briggs Institute methodological guidance for scoping reviews and is reported following the PRISMA-ScR (Preferred Reporting Items for Systematic Reviews and Meta-Analyses extension for Scoping Reviews) guidelines. See **Supplementary Table 1**.

### 2.2 Protocol and registration

A formal protocol was not prospectively registered, which is consistent with scoping review methodology; however, eligibility criteria and methods were defined *a priori* and applied consistently.

### 2.3 Eligibility criteria

We included peer-reviewed, original studies published in English from January 2018 to May 2025 that addressed the molecular mechanisms underlying carbohydrate metabolism dysregulation in PCOS. Human, animal, and *in vitro* cellular model studies were all eligible. Reviews, case reports, and conference abstracts were excluded. We excluded grey literature because of the high methodological rigor required to assess the validity of molecular research; only peer-reviewed publications give the highest probability of reproducibility and validity of results of such studies. Studies were also excluded if they did not present mechanistic insights (e.g., signaling pathways, molecular interactions, or protein expression changes) pertinent to carbohydrate metabolism in PCOS. We also excluded studies focused solely on genetic associations or inheritance patterns when they did not provide direct mechanistic evidence linking the implicated variants or genes to alterations in carbohydrate metabolism within the study itself. Although such studies may suggest potentially relevant biological pathways, they were outside the scope of this review if the mechanistic interpretation relied primarily on inference or on evidence from external studies that did not fit the inclusion criteria of this review. By narrowing our scope in this way, we aimed to provide a clearer and more in-depth mapping of metabolic pathways supported by direct functional or molecular evidence.

### 2.4 Search strategy

A literature search covering the period from 01.01.2018 to 15.05.2025 was conducted, with the last search re-run on 04.01.2026. The final search update in January 2026 did not identify additional eligible studies. Searches were conducted in PubMed, Embase (Elsevier/Embase.com), and Web of Science Core Collection. No database filters were applied. During screening, we restricted inclusion to English-language and peer-reviewed studies. Searches were applied to title/abstract/keywords with appropriate modifications for each database. Search query for PubMed: (“Polycystic Ovary Syndrome”[mh] OR pcos[tiab] OR “polycystic ovary syndrome”[tiab]) AND (“Insulin Resistance”[mh] OR “Hyperinsulinism”[mh] OR “Glucose Metabolism Disorders”[mh] OR “Glycolysis”[mh] OR “Energy Metabolism”[mh] OR “glucose metabolism”[tiab] OR “carbohydrate metabolism”[tiab] OR “insulin resistance”[tiab] OR hyperinsulin*[tiab] OR “insulin signaling”[tiab] OR “insulin tolerance”[tiab] OR “glucose transporter*”[tiab] OR glut1[tiab] OR glut2[tiab] OR glut3[tiab] OR glut4[tiab] OR glut5[tiab] OR glut6[tiab] OR glut7[tiab] OR glut8[tiab] OR glut9[tiab] OR glut10[tiab] OR glut11[tiab] OR glut12[tiab] OR glut13[tiab] OR glut14[tiab] OR “glucose uptake”[tiab] OR glycolysis[tiab] OR “insulin sensitiv*”[tiab] OR “insulin secret*”[tiab] OR pi3k[tiab] OR akt[tiab] OR foxo*[tiab] OR “insulin receptor*”[tiab] OR irs[tiab] OR INSR*[tiab] OR “glucose production”[tiab] OR “beta cell*”[tiab]) AND (receptor*[tiab] OR pathway*[tiab] OR regulat*[tiab] OR upregulat*[tiab] OR downregulat*[tiab] OR crosstalk[tiab] OR interact*[tiab] OR mechanism*[tiab] OR “Signal Transduction”[mh] OR “Gene Expression”[mh] OR signal*[tiab] OR phosphor*[tiab] OR transcriptom*[tiab] OR proteom*[tiab]) AND (“2018/01/01”[dp]: “2025/05/15”[dp]). Full database-specific strategies for PubMed, Embase, and Web of Science are provided in the appendix.

### 2.5 Study selection

All retrieved records were imported into Rayyan (6). Duplicates were removed. Two reviewers independently screened titles and abstracts against the eligibility criteria. Disagreements were resolved through discussion; if consensus could not be reached, a third, independent reviewer was consulted. Where abstracts were unavailable, full texts were assessed. Articles eligible for full-text review were screened, and conflicts were resolved as above.

### 2.6 Data charting

A data-charting form was developed. First, a pilot form was tested on five studies to ensure reproducibility and determine which data to extract. Two independent reviewers completed the pilot charting, and the form was refined through group discussions and subsequent testing until consensus and 90% reproducibility were reached. The finalized form was then distributed to reviewers for systematic data extraction. Two independent reviewers extracted following data: title, authors, year, journal, model: human/animal, type of study, country (countries) where the study was conducted, aim of study, summary of methodology, information about sample group (number of women with PCOS vs controls), and PCOS diagnostic criteria (applicable to human model), species, strain, sex, number of subjects and PCOS induction (applicable to animal model studies), cell tissue origin (applicable to *in vitro* studies), statistical methods used, laboratory methods and techniques used, described mechanism/pathway of carbohydrate metabolism dysregulation, limitations. See **Supplementary Table 2** for data extraction and details of studies. A scoping approach is appropriate to map heterogeneous evidence. Consistent with common scoping practice, we did not undertake a formal critical appraisal.

### 2.7 Synthesis of results

Results were organized according to the primary tissues or cell types analyzed in each study. Because several studies investigated interactions across multiple tissues, a separate category addressing systemic or general alterations in glucose metabolism was included. Findings were synthesized using both tabular and narrative formats, summarizing molecular alterations within each tissue or cell type. The main tissues and systems analyzed were granulosa cells and ovarian tissue, endometrium, liver, adipose tissue, skeletal muscle, and systemic regulatory pathways. Alterations in glucose metabolism were grouped according to affected receptors, enzymes, signaling cascades, and relevant environmental or endocrine modulators.

## 3 Results

### 3.1 Summary of search results

**Figure 1:**
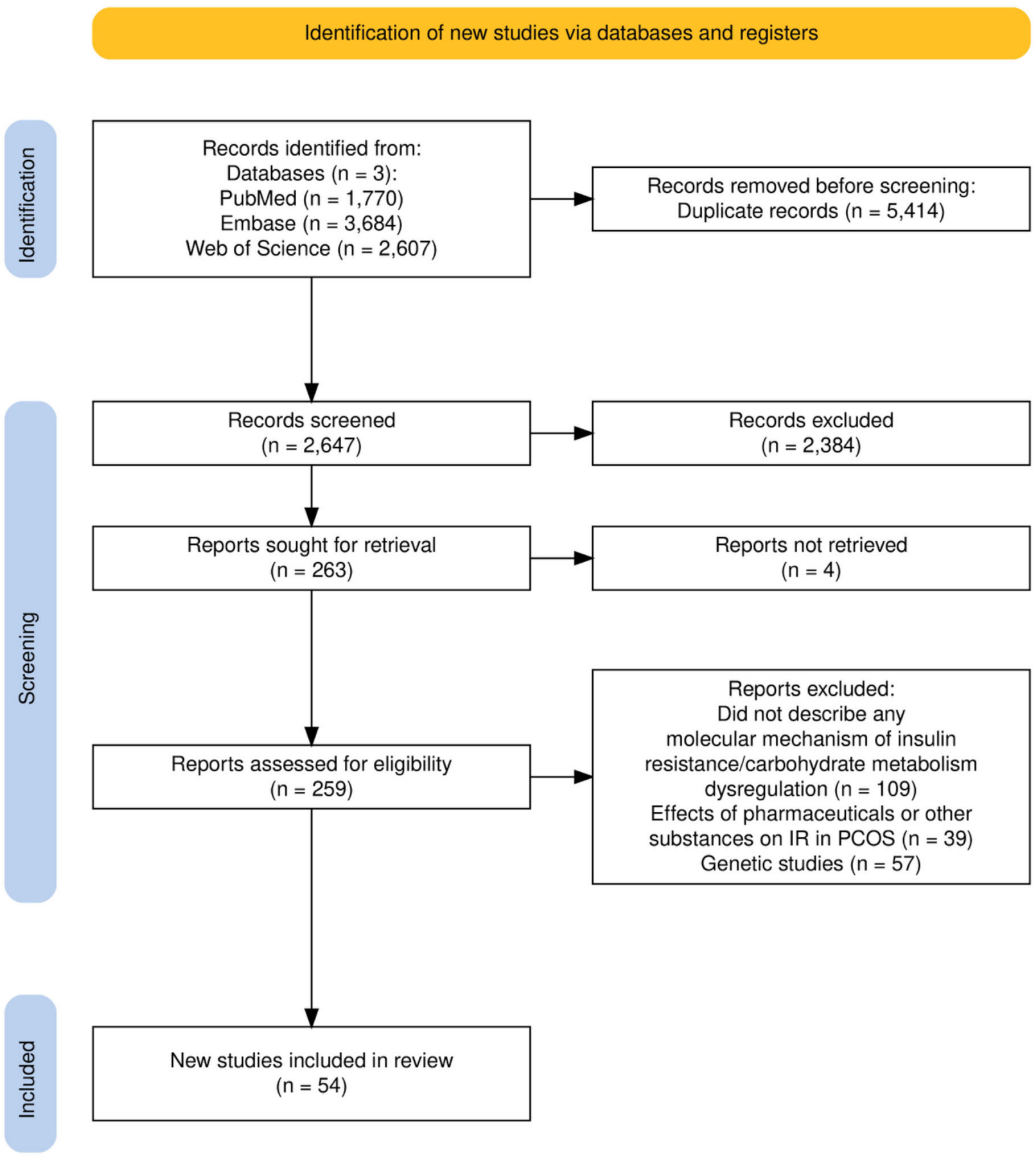
PRISMA flow diagram illustrating the identification, screening, eligibility assessment, and inclusion of studies in the scoping review.

Overall, the electronic search of the databases returned 8061 records. After removing duplicates, 2647 records remained. After title and abstract screening, 263 records were selected and sought for retrieval; 4 studies could not be accessed, and 259 underwent full-text screening. 54 studies (**Supplementary Table 3**) met the inclusion criteria and were presented in the review (**Figure 1**) (7).

#### 3.1.1 Summary of included studies (characteristics of sources of evidence)

**Figure 2:**
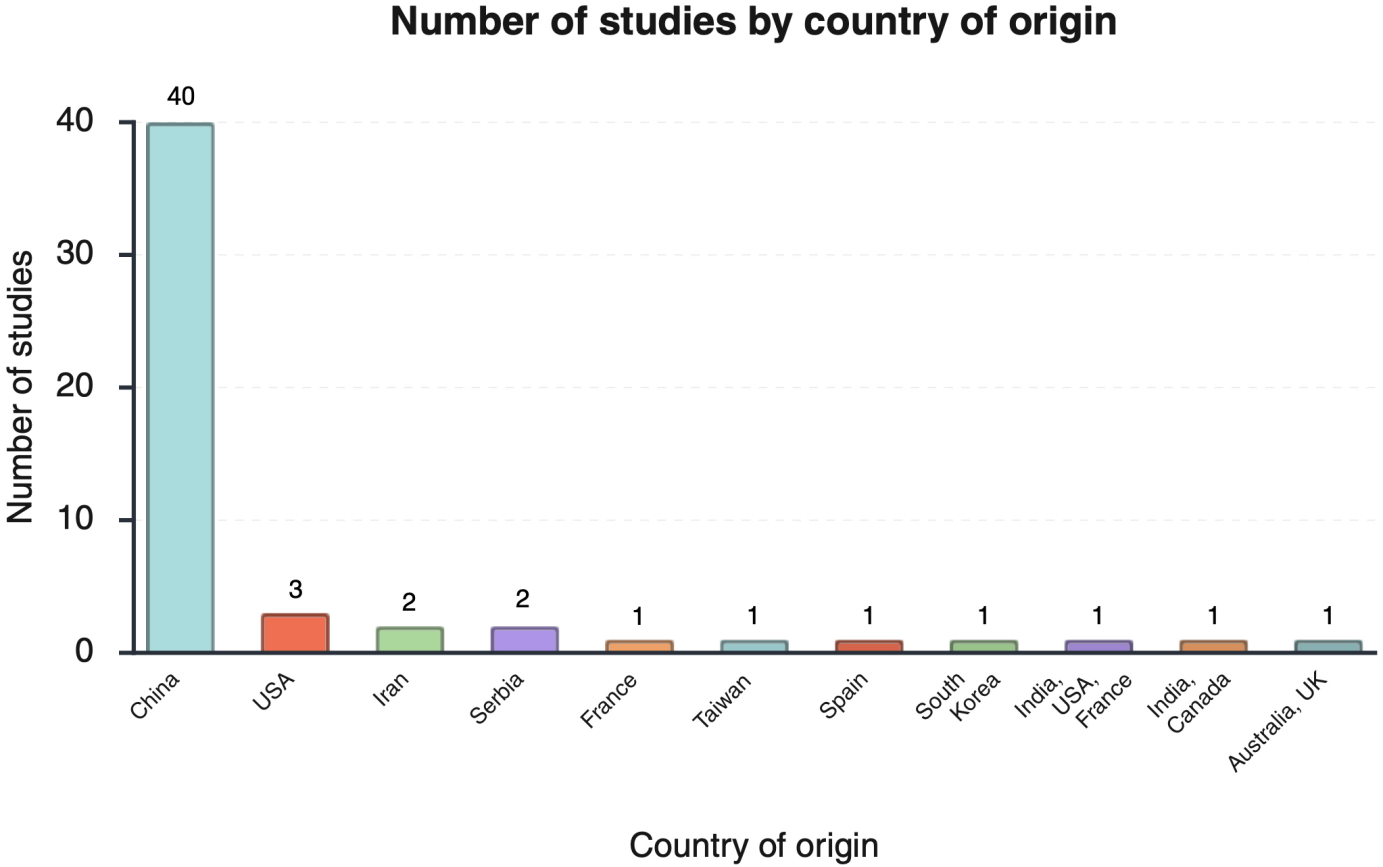
Distribution of included studies by country of origin. Created in https://BioRender.com.

**Figure 3:**
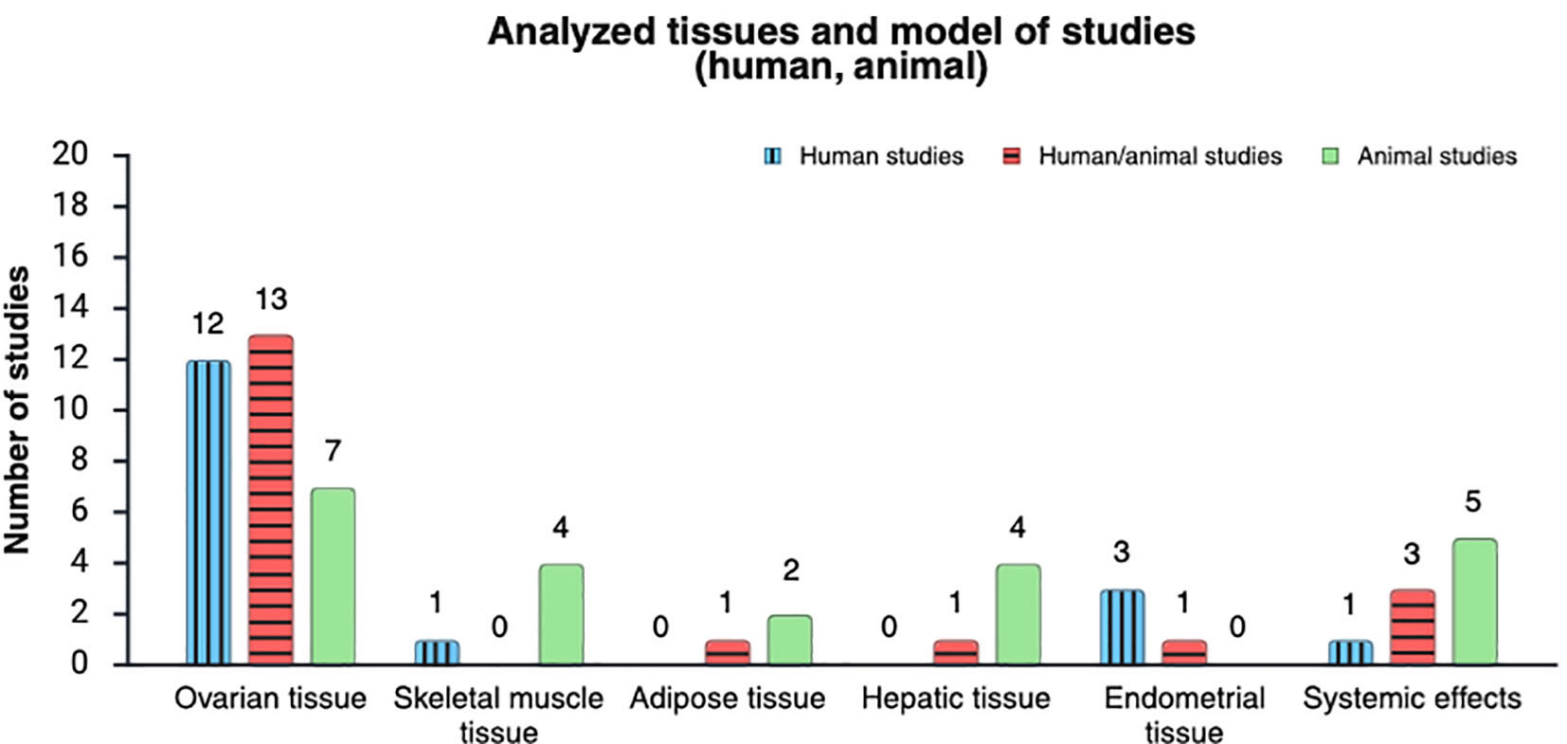
Distribution of analyzed tissues across included studies by model (human, animal). Created in https://BioRender.com.

A total of 54 studies were included. Most evidence originated from China (n=40), with additional studies conducted in the USA (n=3), Iran (n=2), and single studies from France (n=1), Taiwan (n=1), Spain (n=1), South Korea (n=1), and Serbia (n=2). Several studies were conducted across multiple countries, including India-USA-France (n=1), India-Canada (n=1), and Australia-UK (n=1) (**Figure 2**). Across included studies, evidence was mainly from combined human-and-animal approaches (n=19), slightly less represented were human-only studies (n=17) and animal-only studies (n=18) (**Figure 3**). Methodological approaches were frequently mixed within studies: *in vitro* methods were reported in 36 studies, *in vivo* analyses in 37, ex vivo analyses in 25, and in silico analyses in 20. Among epidemiologic/observational designs, cross-sectional studies (n=11) and case–control studies (n=20) were reported. Among human studies that reported PCOS diagnostic criteria, the 2003 Rotterdam criteria were most commonly used (n=29), with one study using the 2018 PCOS Evaluation and Management guideline, one reporting Rotterdam criteria without specifying the version, and one not reporting diagnostic criteria. Animal models most commonly involved C57BL/6 mice (n=15) and Sprague Dawley rats (n=14), with additional models including Wistar rats (n=3), wild-type mice (n=1), Holtzman strain rats (n=1), and unspecified rat strains (n=1). The most frequently studied tissue sources were granulosa cells (n=25 studies) and ovarian tissue (n=7 studies) (total ovarian-focused sources n=31). Other tissues/cell types included hepatic tissue (n=9 studies), skeletal muscle tissue (n=5 studies), endometrial cells (n=5 studies), adipose tissue (n=5 studies), intestinal tissue (n=1 study), insulinoma cell line (n=1 study), bone marrow cells (n=1 study), and pituitary cells (n=1 study) Multiple tissues could be examined within a single study. Publication years ranged from 2018 to 2025, with the largest number published in 2021 (n=11); other yearly counts were 2018 (n=10), 2019 (n=3), 2020 (n=9), 2022 (n=10), 2023 (n=5), 2024 (n=4), and 2025 (n=2). Below is the narrative summary of the included sources of evidence.

### 3.2 Granulosa cells

#### 3.2.1 Changes in the MAPK signaling

In PCOS, the activity profile of the mitogen-activated protein kinase (MAPK) family is altered, which is believed to contribute to IR. Studies on human granulosa cells (hGCs) from women with PCOS revealed that one of the adaptor proteins, Src-associated in mitosis 68 kDa (Sam68), impairs the MAPK/extracellular signal-regulated kinase (ERK) pathway in response to insulin. This impairment reduces ERK1/2 activity, leading to lower levels of phosphorylated ERK1/2 (8), despite increased total ERK1/2 abundance. The reduction in p-ERK1/2 decreases the expression of insulin receptor (INSR) and insulin receptor substrate 1 (IRS1), promoting IR in hGCs (9).

Experiments on ovarian tissue from PCOS rats also indicate alterations in stress-responsive MAPK branches. c-Jun N-terminal kinase (JNK) and p38, along with elevated levels of their phosphorylated forms, have been associated with higher ovarian macrophage migration inhibitory factor (MIF) expression. This is believed to promote IR, although the study did not directly measure other components of the insulin signaling cascade (10).

#### 3.2.2 Changes in the upstream components of PI3K/AKT

##### 3.2.2.1 INSR/IRS signaling

Proximal insulin signaling in granulosa cells appears to be disrupted at the level of both IRS1 and IRS2. In hGCs, the insulin receptor beta-subunit (INSRbeta) is downregulated, and inhibitory phosphorylation of IRS1 at Ser307 (pIRS1-Ser307) is increased (9). IRS1 activity is further modulated by 11beta-hydroxysteroid dehydrogenase type 1 (HSD11B1), which is elevated in both human and rat PCOS ovaries. Although the underlying molecular mechanism has been characterized primarily in rats, increased *HSD11B* and *Hsd11b1* mRNA expression is observed in both species and is consistent with enhanced local cortisol production, which (in rat ovaries) has been shown to reduce IRS1 levels. In line with this, the selective HSD11B1 inhibitor BVT-2733 restores IRS1 toward control levels and improves insulin tolerance (11). AMP-activated protein kinase (*AMPK*) knockdown in PCOS-like KGN cells also downregulates HSD11B1, suggesting a link between compromised AMPK signaling and PCOS (12). Additional negative regulators of IRS1, which are shown to reduce its protein abundance, include miR-103 in rat granulosa cells (GCs) and high mobility group box 1 (HMGB1), a damage-associated molecular pattern molecule whose concentration is elevated in hGCs from patients with PCOS (13, 14).

Adipokines like chemerin also seem to be involved in IRS signaling disruption. Patients with both PCOS and IR exhibited significantly higher chemerin concentrations in follicular fluid and plasma compared to controls. hGCs of these patients exhibit increased chemerin and chemokine-like receptor 1 (CMKLR1) expression, while the levels of G protein–coupled receptor 1 (GPR1) and C-C motif chemokine receptor–like 2 (CCRL2) (other chemerin receptors) remained unchanged. Notably, chemerin pretreatment in hGCs led to a significant enhancement of insulin-stimulated IRS1 Ser307 phosphorylation (inhibitory) and attenuation of insulin-stimulated phosphorylation of IRS1/2 Tyr612 and protein kinase B (AKT) Ser473 (activating). However, in hGCs, chemerin did not alter insulin-stimulated INSRbeta Tyr1150/1151 phosphorylation (15).

**Figure 4:**
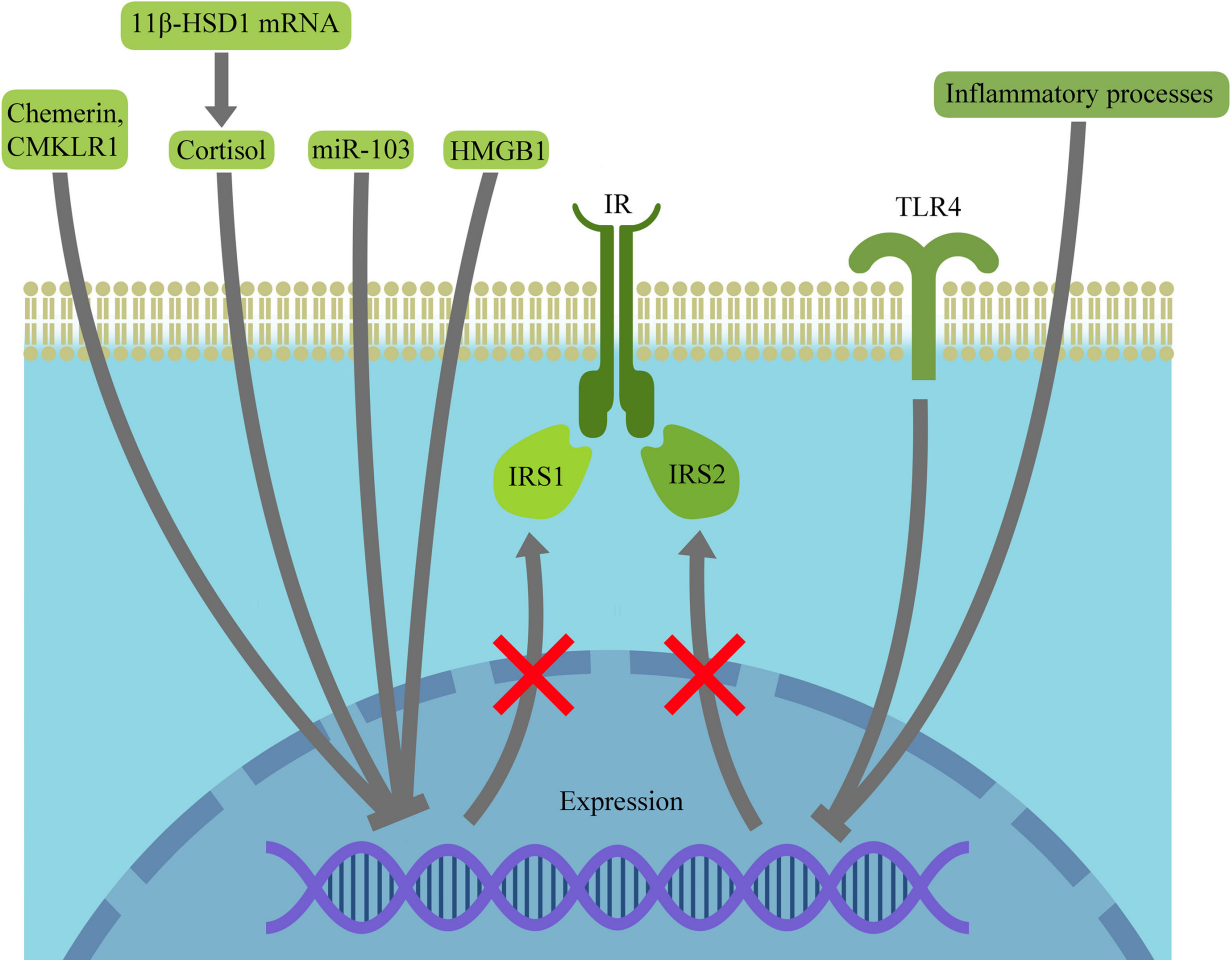
Upstream defects of insulin signaling contributing to impaired PI3K/AKT pathway activity in granulosa cells in PCOS. Multiple endocrine, inflammatory, and adipokine-related factors converge on proximal insulin signaling in granulosa cells. Increased HSD11B1 and local cortisol production, miR-103, HMGB1, chemerin signaling through CMKLR1, and inflammatory TLR4 signaling are associated with reduced expression or impaired function of IRS1/IRS2, thereby weakening signaling from the insulin receptor to the PI3K/AKT pathway.

IRS2 also appears to be suppressed in PCOS through both endocrine and inflammatory mechanisms. Elevated luteinizing hormone (LH) levels and LH pulse frequency contribute to dysregulation of follicle-stimulating hormone (FSH)-dependent glucose uptake and glycogen synthesis in PCOS (16). In non-PCOS granulosa cells, FSH activates follicle-stimulating hormone receptor (FSHR) and the IRS2-dependent insulin signaling pathway by increasing IRS2 tyrosine phosphorylation and decreasing serine phosphorylation. This, in turn, inactivates glycogen synthase kinase 3 beta (GSK3beta), increases expression/activity of phosphatase 1 and glucose synthase, thereby enhancing glucose uptake and glycogenesis. In rats, the presence of an LH analogue such as human chorionic gonadotropin (hCG) alongside FSH inhibits FSH-stimulated glucose uptake and glycogen synthesis, partly through downregulation of FSH-induced *Irs2* mRNA expression. A similar inhibitory effect is observed in HEK293 cells co-expressing FSH and LH receptors, where higher hCG concentrations reduce FSH-stimulated glucose uptake and promote receptor heteromerization (17). Inflammatory pathways may also contribute to IRS2 suppression. Experiments on rats show Toll-like receptor 4 (TLR4) upregulation in PCOS rat follicles, whereas silencing Tlr4 with siRNA increases IRS2 levels, indicating that TLR4 activation may inhibit IRS2 and thereby disrupt insulin signaling (18). Serum amyloid A1 (SAA1) has likewise been linked to GC IR through TLR2/4-dependent activation of the nuclear factor kappa B (NFkappaB) pathway (19). In contrast to this predominantly suppressive pattern, He et al. reported significant overexpression of IRS1 and insulin-like growth factor 1 receptor (IGF-1R) in hGCs (20). Collectively, these findings indicate that endocrine, inflammatory, and adipokine-related abnormalities in PCOS converge on IRS dysregulation, thereby impairing proximal insulin signaling in GCs (**Figure 4**).

##### 3.2.2.2 Changes in PI3K/AKT signaling and GLUT4 trafficking

Phosphoinositide 3-kinase (PI3K)/AKT is a central pathway in insulin signal transduction, with glucose transporter type 4 (GLUT4) acting as a key downstream effector. A substantial body of evidence indicates that impaired PI3K/AKT signaling contributes to IR in PCOS. One mechanism implicated in this process involves KLOTHO. In ovarian tissue from PCOS rats, KLOTHO protein expression is elevated, whereas its miRNA regulators, miR-29a-5p and miR-126-5p, are significantly reduced. Increased KLOTHO levels are associated with lower IGF-1R protein abundance and diminished downstream signaling, particularly within the AKT pathway. Consistent with this, AKT phosphorylation at Ser473 and Thr308 is markedly reduced in GCs from patients with PCOS and in ovarian tissue from PCOS rats (21).

Additional mechanisms may further impair AKT activation. In PCOS mice, overexpression of the skeletal muscle- and kidney-enriched inositol phosphatase (SKIP), driven by m^6^A hypermethylation of *Skip* mRNA, promotes dephosphorylation of phosphatidylinositol (3–5)-trisphosphate (PIP3), thereby limiting AKT activation and disrupting insulin signaling. Elevated expression of the m6A “readers” YTHDF3 and HNRNPA2B1 has also been observed (22). Further factors diminishing insulin-induced AKT phosphorylation include activating transcription factor 4 (ATF4) in human studies and C-C motif chemokine ligand 5 (CCL5) in PCOS mouse models (23, 24), as well as PH domain leucine-rich repeat protein phosphatase 1 (PHLPP1), which inhibits AKT activation in human ovarian GCs under hyperandrogenic conditions (25). Similarly, miRNAs such as miR-133a-3p are upregulated in GCs from patients with PCOS, particularly those with obesity, and are associated with decreased p-AKT and PI3K levels (26). Increased ovarian HSD11B1 activity is also linked to reduced AKT Ser473 phosphorylation in rat ovaries (11).

Furthermore, AKT activity appears to be modulated by interactions between Casitas B-lineage lymphoma protein (CBL)-associated protein (CAP) and Lck/Yes novel tyrosine kinase (LYN). The Sorbin and SH3 Domain Containing 1 (*SORBS1)* gene is a parent gene of the CAP, which is linked with the insulin receptor. *SORBS1* is downregulated in granulosa cells from insulin-resistant patients with PCOS. When CAP’s SH3 domain is bound to CBL’s proline-rich region domain, CT10 regulator of kinase II (CRKII)-mediated membrane GLUT4 translocation and AKT Thr308 phosphorylation are enhanced, but in contrast, when LYN’s SH3 domain is bound to CBL’s proline-rich region domain, NFkappaB-mediated inflammatory marker expression is enhanced, but CRKII-mediated membrane GLUT4 translocation or AKT Thr308 phosphorylation are not significantly affected. PCOS mice exhibit improvement in IR and GLUT4 translocation after *Cap* overexpression, *Cap*-transactivating metformin therapy, and by promoting *Cbl-CrkII* binding (via Cbl3YF phospho mutant overexpression). It is proposed that *Cap* downregulation and inhibition of AKT Thr308 signaling and GLUT4 membrane trafficking could contribute to IR in PCOS mice (27).

Decreased GLUT4 expression is also suggested to be linked to IR in PCOS. A study by Froment et al. showed that knockout of the *alpha1-AMPK* gene in KGN cells is associated with decreased GLUT4 expression. It is further speculated that lower alpha1-AMPK activity may be associated with PCOS, and that AMPK is the main kinase activated by metformin therapy (12). Additionally, GCs from patients with PCOS and IR exhibit increased *SAA1* mRNA levels, further supporting their role in SAA1 production. Both interleukin-1beta (IL-1beta) and SAA1 itself have been shown to elevate SAA1 levels in cultured GCs, indicating a feedforward loop of *de novo* SAA1 synthesis. Functionally, SAA1 increases phosphatase and tensin homolog (PTEN) abundance in GCs, attenuates insulin-induced AKT phosphorylation, and impairs insulin-stimulated GLUT4 translocation from the cytoplasm to the cell membrane, leading to reduced glucose uptake (19). Another inflammatory molecule elevated in PCOS is HMGB1, which also decreases AKT phosphorylation and reduces GLUT4 translocation (14).

*In vitro* experiments have shown that high androgen concentrations suppress Gene associated with Retinoid–IFN-induced Mortality-19 (GRIM19) expression in an androgen receptor (AR)-dependent mechanism. GRIM19 deficiency leads to abnormal glucose metabolism by suppressing the Ras-related C3 botulinum toxin substrate 1 (RAC1)/GLUT4 pathway, ultimately reducing insulin-stimulated glucose uptake in KGN cells (28).

#### 3.2.3 Changes in glycolysis and cellular energy metabolism dysfunction

##### 3.2.3.1 Altered glycolytic activity

PCOS is associated with dysregulated glycolysis and altered cellular energy metabolism in GCs. Overall, in PCOS hGCs, glycolytic function is compromised. Key glycolytic enzymes (hexokinase-1, platelet phosphofructokinase, muscular phosphofructokinase, and muscular pyruvate kinase) are significantly downregulated at both mRNA and protein levels compared to controls. Correspondingly, GC lactate production (as a proxy for glycolysis) is significantly lower in patients with PCOS. GCs also exhibit diminished ATP levels, indicating impaired energy conversion. Compromised mitochondrial function is attributed to decreased mitochondrial membrane potential (Δψm) (29). Reduced GRIM19 is one of the factors proposed to contribute to mitochondrial and glycolytic dysregulation. A model based on KGN cells with reduced GRIM19 expression exhibited decreased glycolysis, reduced mitochondrial membrane potential and mitochondrial complex I activity, inhibition of mitochondrial respiration, and decreased ATP production (28). Similarly, hexokinase domain-containing protein 1 (HKDC1), an enzyme involved in glycolysis and gluconeogenesis, has also been indicated to impair glucose utilization in PCOS granulosa cells. Expression of this protein is decreased in hGCs. Possible effects of its downregulation have been studied in KGN cells, in which *HKDC1* silencing caused mitochondrial abnormalities, decreased mitochondrial membrane potential (Δψm), reduced glucose consumption, decreased glucose-6-phosphate formation, and lowered lactate output, indicating impaired glycolytic flux (30).

##### 3.2.3.2 PGK1 upregulation, androgen receptor signaling, and other mitochondrial alterations

Further alterations in the glycolytic pathway of hGCs involve phosphoglycerate kinase 1 (PGK1), which has been identified as an AR-interacting protein and, unlike other glycolytic enzymes, appears elevated. GCs from patients with PCOS showed higher mRNA and protein levels of PGK1 and AR than control samples. In a dehydroepiandrosterone (DHEA)-induced PCOS-like mouse model, PGK1 and AR were likewise significantly increased in ovarian tissue. PGK1 catalyzes a key step in glycolysis and has been shown to convert more glucose into lactate in hGCs, thereby increasing the glycolysis rate. Mechanistically, PGK1 reduces AR’s ubiquitination by engaging the E3 ubiquitin ligase S-phase kinase–associated protein 2 (SKP2), thereby increasing AR protein stability. PGK1 also promotes nuclear translocation of AR in a manner dependent on androgen stimulation. Transcriptomic profiling (RNA-sequencing) revealed that *PGK1* knockdown reduced the expression of numerous AR-dependent genes, including *MAP2K6*, thereby enhancing glycolysis (31). Moreover, *CCNL* (a long noncoding RNA) overexpression in KGN cells and hGCs disrupted mitochondrial function, as evidenced by higher mitochondrial reactive oxygen species and lower ATP levels compared with controls; ATP was also reduced in women with PCOS. Collectively, these data implicate *CCNL* overexpression in mitochondrial dysfunction (32).

#### 3.2.4 Changes in FOXO transcription factors

##### 3.2.4.1 FOXO3

Forkhead Box O (FOXO) is a group of transcription factors that are downstream regulators of the insulin pathway (33). Their increased activity is suggested to contribute to IR in PCOS GCs. One of the factors, FOXO3, is linked to IR in GCs from patients with PCOS. Its mRNA and nuclear expression are elevated and associated with IR parameters (34). FOXO3 has been shown to be negatively correlated with miR-29c-3p; while women with PCOS exhibit low miR-29c-3p levels but high FOXO3 levels in peripheral blood. Upon silencing of miR-29c-3p in KGN cells, FOXO3 levels were significantly elevated. Studies on a rat model further confirmed these claims and found a significant association with decreased miR-29c-3p and IR. Furthermore, bioinformatic analysis identified a potential binding site for miR-29c-3p within the 3’ untranslated region of *FOXO3* mRNA in KGN cells (35). FOXO3 is further regulated by SH2B adaptor protein 3 (LNK), whose levels are significantly elevated in GCs of patients with PCOS. KGN cells with artificially overexpressed LNK presented with inhibited phosphorylation of AKT and FOXO3, nuclear localization of FOXO3, and upregulation of apoptotic markers. These effects were reversed following LNK knockdown. Consistent with these findings, a PCOS mouse model with LNK knockout showed substantially elevated GLUT4 levels and improved glucose metabolism compared with wild-type PCOS mice (34).

##### 3.2.4.2 FOXO1

*FOXO1* is regulated by *CCNL*, a long noncoding RNA (lncRNA), which is overexpressed in GCs from patients with PCOS and those with higher Homeostatic Model Assessment - Insulin Resistance (HOMA-IR) values. The expression levels of key proteins involved in glucose uptake, such as IRS1 and GLUT4, are decreased when *CCNL* is overexpressed, thereby impairing glucose metabolism in GCs. It is suggested that *CCNL* impairs glucose uptake by raising *FOXO1* expression because all effects caused by *CCNL* overexpression are rescued by *FOXO1* knockdown. *CCNL* was also shown to bind to *FOXO1*, indicating that it may interact with and regulate its activity (32). Moreover, studies point to other factors involved in FOXO1 upregulation; for instance, FOXO1 was also shown to be elevated by increased levels of angiopoietin-like 2 (ANGPTL2) in GCs (36). Increased expression of miR-133a-3p also increased p-FOXO1 levels, accompanied by increased INSR, decreased GLUT4 levels, and decreased p-GSK3beta levels (37).

#### 3.2.5 Changes to other miRNAs and IR in GCs

Other miRNAs, such as miR-106a-5p and miR-155-5p, also appear to be differentially expressed, along with their target genes. They are linked to inflammation and insulin sensitivity. GCs of women with PCOS exhibit much lower expression of miR-106a-5p and miR-155-5p. The downregulation of miR-106a-5p and miR-155-5p aligns with the upregulation of their target genes—*Stat3, Cd28, Gsk3b*, and *Nr1h3*—in prenatally androgenized mice compared to controls. These genes are associated with PCOS development and connected to the insulin signaling pathway (38). Likewise, miR-146-5p is downregulated and is also linked to metabolic dysfunction, potentially contributing to increased IR in PCOS rat ovaries (37).

#### 3.2.6 Effects of plasticizers on insulin signaling in granulosa cells

Women with PCOS exhibit higher levels of bisphenol A (BPA) in their serum compared to women without PCOS, with no differences in urine. BPA is also shown to significantly exacerbate the DHEA-induced metabolic IR phenotype in mice. In hGCs, BPA activates the aryl hydrocarbon receptor (AHR) and promotes its nuclear translocation from the cytosol, consequently reducing cell viability and impairing glucose uptake. Combining this plasticizer with an AHR inhibitor reverses these changes, indicating AHR-dependent IR. Data show that BPA promotes AHR binding to a suppressor of the GLUT4 genes, resulting in a significant drop in its expression (39). Similarly, phthalates modulate the insulin signaling cascade (EGFR/PI3K/AKT/STAT3/SRC), indicating that endocrine disruptors can access the same signaling pathways as androgens, though through different mechanisms. In one study, exposure to dihydrotestosterone (DHT), dibutyl phthalate (DBP), or di(2-ethylhexyl) phthalate (DEHP) resulted in the upregulation of *AKT1* and *PIK3R1*. *AKT1* expression increased approximately sevenfold in the DHT group and five- to sixfold in the DBP and DEHP groups, respectively. *PIK3R1* expression followed the same trend, increasing two- to fourfold in the presence of DBP or DEHP. Simultaneous elevations in *SRC, EGFR*, and *STAT3* mRNA indicated activation of a complex signaling axis that integrated downstream insulin signaling, growth factor signaling, and inflammatory pathways. Together, these results imply that plasticizers such as phthalates can modulate essential insulin-related signaling nodes in GCs, rather than acting through nonspecific toxicity alone (40).

### 3.3 Changes in skeletal muscle

Skeletal muscle IR in PCOS appears to arise from a combination of mitochondrial dysfunction, altered insulin signaling, and extracellular matrix remodeling. In line with this, a study in a PCOS mouse model showed a disrupted NAD^+^/NADH ratio in skeletal muscle associated with IR. Both the NAD^+^/NADH ratio and ATP content were significantly decreased, whereas absolute NAD^+^ levels were unchanged, consistent with mitochondrial impairment. In the same model, a shift in muscle fiber composition toward a reduced proportion of slow-twitch (type I) fibers relative to fast-twitch (type II) fibers further contributes to skeletal muscle IR. Because type I fibers are rich in mitochondria and have high oxidative capacity, a lower proportion of these fibers is likely to exacerbate IR. Moreover, the authors propose that mitochondrial dysfunction, rather than intramyocellular lipid accumulation, is the primary driver of skeletal muscle IR in PCOS, as no lipid accumulation was detected in the skeletal muscle of PCOS mice (41). Mitochondrial dysfunction in this model appears to be further promoted by increased activation of mechanistic target of rapamycin complex 1 (mTORC1). DHEA-treated prepubertal mice with a PCOS-like phenotype exhibit basal mTORC1 hyperphosphorylation in skeletal muscle, which is associated with reduced GLUT4 abundance at the plasma membrane, mitochondrial dysfunction, and diminished AKT phosphorylation in response to insulin; together, these defects are thought to contribute to skeletal muscle IR. In addition, autophagy, which appears to play a protective role against IR in skeletal muscle, is reduced in PCOS mice, thereby further aggravating IR and mitochondrial impairment (42). In contrast to these rodent data showing basal mTORC1 overactivation, findings from female patients with PCOS indicate impaired insulin-stimulated mechanistic target of rapamycin (mTOR) phosphorylation. This apparent discrepancy may reflect differences in species, obesity status, and the distinction between basal mTORC1 activity in experimental PCOS models and insulin-stimulated mTOR phosphorylation in human skeletal muscle. In the human study, among the four groups (PCOS with obesity, PCOS–lean, non-PCOS with obesity, non-PCOS–lean), impaired insulin-stimulated mTOR phosphorylation was observed only in women with PCOS and persisted even after 12 weeks of aerobic exercise training. In women with PCOS and obesity, genes in the transforming growth factor beta (TGFbeta) signaling pathway associated with fibrosis are upregulated in skeletal muscle. Specifically, genes encoding extracellular matrix (ECM) components (*COL1A2, COL3A1*), enzymes involved in collagen crosslinking and deposition (*LOX, DCN*), and ligands and receptors such as *TGFB2* and *TGFBR2* are elevated. Although exercise training does not fully reverse these fibrosis-related gene expression changes, some genes, including *COL1A2, COL3A1, DCN*, and *LOX*, display altered regulation after an exercise session. Twelve weeks of aerobic exercise improved insulin sensitivity in women with PCOS and obesity, but did not completely normalize insulin signaling. While exercise increased phosphorylation of Akt substrate of 160 kDa (AS160), it also produced unexpected changes, such as reduced phosphorylation of AKT and GSK3. Furthermore, changes in insulin sensitivity (glucose infusion rate) were negatively correlated with the Free Androgen Index, suggesting that reductions in hyperandrogenism may improve insulin sensitivity in PCOS. However, Free Androgen Index does not correlate with phosphorylation of insulin signaling proteins or with fibrosis-related gene expression, indicating that hyperandrogenism alone does not fully explain the observed molecular alterations. On this basis, it is suggested that reduced insulin-stimulated mTOR phosphorylation in PCOS may represent a key mechanism of intrinsic skeletal muscle IR. Upregulation of the TGFbeta pathway and ECM remodeling may also contribute to IR by promoting a pro-fibrotic environment that impairs insulin signaling and, potentially, glucose uptake. The elevated expression of TGFbeta ligands, receptors, and ECM components suggests that PCOS skeletal muscle is predisposed to fibrosis, which could interfere with normal insulin action. Moreover, TGFbeta ligand signaling via SMAD–mTOR and SMAD–AKT pathways, together with tissue fibrosis, may be particularly important in driving IR in PCOS, especially in the presence of obesity and relative resistance to lifestyle interventions. Unlike women with obesity and without PCOS, those with PCOS did not show significant and sustained reductions in fibrosis-related gene expression after exercise, suggesting that ECM remodeling in PCOS may be relatively resistant to exercise (43). Additional alterations in the IRS/PI3K/AKT pathway in skeletal muscle have also been demonstrated. In overfed PCOS rats, increased IRS1 phosphorylation at Ser307 and reduced AKT phosphorylation at Ser473 indicate impaired insulin-mediated glucose uptake. In contrast, total AKT protein levels and phosphorylation at Thr308 remained unchanged, suggesting selective dysregulation of specific nodes within the insulin signaling cascade. Moreover, AMPK phosphorylation at Thr172 was elevated, likely as a compensatory response to metabolic stress. DHT further exacerbates metabolic dysfunction by reducing GLUT4 protein levels in skeletal muscle, thereby limiting glucose uptake. To compensate for impaired glucose metabolism, skeletal muscle shifts toward mitochondrial beta-oxidation of fatty acids, as evidenced by increased expression of *Fatp1* and *Cpt1b* in the overfed + DHT group; however, this shift was accompanied by oxidative stress, as indicated by elevated malondialdehyde levels (44). It is important to note that most of the studies described above were conducted in rodents with free access to food, overfed rodents, or women with obesity and PCOS. In contrast, lean DHT-treated mice have been reported to exhibit elevated insulin-stimulated glucose uptake in skeletal muscle compared with non-PCOS control mice, accompanied by increased AKT phosphorylation and higher expression of key insulin signaling proteins, including GLUT1, GLUT4, INSRbeta-subunit, and IRS1/2. The authors proposed that enhanced insulin signaling in skeletal muscle may represent a compensatory mechanism in response to IR in the liver and white adipose tissue (WAT). An alternative explanation is that obesity exacerbates skeletal muscle insulin dysfunction, whereas in lean individuals, hyperandrogenemia preferentially impairs glucose metabolism in the liver and WAT (45).

### 3.4 Changes in adipose tissue

In adipocytes exposed to high glucose and insulin, similar to what is observed in women with PCOS, the expression of miR-33b-5p is increased. This elevation leads to decreased expression of *GLUT4*, high mobility group AT-hook 2 (*HMGA2*), and sterol regulatory element-binding transcription factor 1 (*SREBF1*). miR-33b-5p directly inhibits HMGA2 and indirectly suppresses SREBF1, both of which are positive regulators of GLUT4 expression. As a result, glucose uptake in adipocytes is impaired, promoting IR (46). In a postnatally overfed PCOS rat model treated with DHT, insulin sensitivity, assessed by levels of IRS1, p-AKT, and phosphorylated p-ERK1/2, was maintained in visceral adipose tissue (VAT) but deteriorated in subcutaneous adipose tissue. Furthermore, preserved insulin sensitivity in VAT correlated with AMPK activation, suggesting that AMPK may help maintain tissue-specific metabolic functions under overfeeding conditions. Conversely, subcutaneous tissue showed reduced AMPK activity along with disrupted fatty acid turnover, evidenced by altered expression of genes involved in lipogenesis, such as those encoding acetyl-CoA carboxylase, fatty acid synthase, and stearoyl-CoA desaturase 1, as well as phosphoenolpyruvate carboxykinase (PEPCK). These changes likely contribute to the expansion of VAT and a decline in overall insulin sensitivity (47).

### 3.5 Changes in hepatic tissue

Hepatic glucose metabolism is also markedly altered in PCOS, and accumulating evidence indicates that IR in the liver results from a combination of inflammatory activity, androgen excess, disturbances in the regulation of glycolytic and gluconeogenic enzymes, and impaired intracellular signaling dynamics. In rodent models of PCOS, hepatic inflammation plays a central role: markedly elevated C-reactive protein (CRP) is associated with impaired insulin signaling, increased gluconeogenic drive, and reduced insulin sensitivity. When CRP is genetically eliminated, the liver exhibits improved insulin responsiveness, reflected by increased phosphorylation of the INSR and AKT, reduced expression of the gluconeogenic enzyme PEPCK, and significantly higher glucose infusion and disposal rates, together with lower hepatic glucose production (48). These findings highlight CRP as an important inflammatory mediator maintaining a gluconeogenic, insulin-resistant hepatic phenotype in PCOS.

**Figure 5:**
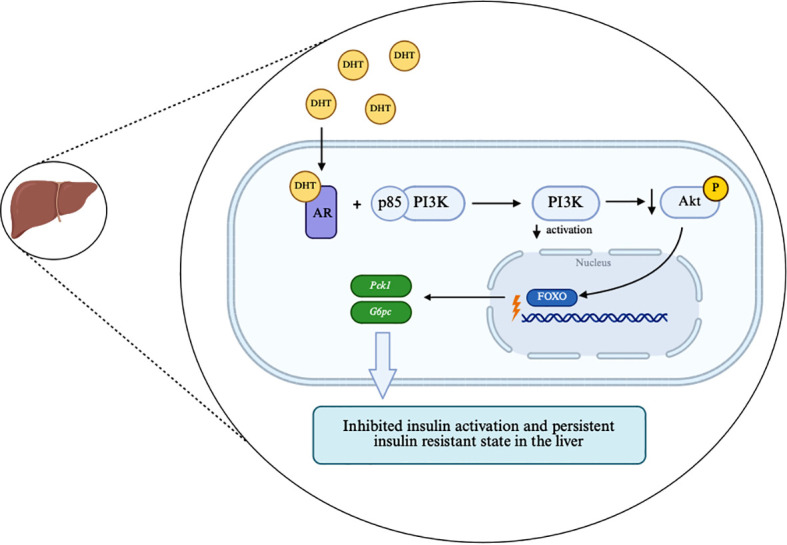
Androgen receptor–mediated impairment of insulin signaling in hepatic tissue in PCOS. Elevated DHT activates hepatic androgen receptor AR, which interacts with the p85 subunit of PI3K, limiting PI3K activation and reducing AKT phosphorylation. Reduced AKT activity allows FOXO1 to remain transcriptionally active and sustain expression of *Pck1* and *G6pc*, thereby promoting hepatic gluconeogenesis and a persistent insulin-resistant state. Created in BioRender. Sas, O. (2026) https://BioRender.com/cyczhmd.

Additional inflammatory mechanisms appear to further compromise hepatic metabolism. Neutrophil extracellular traps (NETs), which are elevated in PCOS models, accumulate within hepatic tissue, where they impair insulin signaling and glycolytic function. Their presence is associated with reduced AKT and GSK phosphorylation and lower hepatic GLUT4 protein abundance. At the transcript level, NET exposure is accompanied by decreased expression of *Irs1*, *Irs2*, *Glut2*, and glycolysis-related genes, including *Pfkfb1*, *Pfkfb3*, *Pfk*, and *Ldha* (49). Although transcriptional changes in some glycolytic genes did not reach statistical significance in rat liver, the overall pattern suggests that neutrophil extracellular traps suppress hepatic glycolysis and reduce insulin responsiveness, leading to metabolic inflexibility. Evaluation of metabolic patterns further highlights extensive changes in hepatic carbohydrate metabolism. Liver tissue from PCOS mice shows upregulated gluconeogenesis, evidenced by elevated levels of intermediates such as phosphoenolpyruvate and pyruvate, alongside reduced levels of tricarboxylic acid cycle intermediates, pointing to impaired glucose metabolism (50). Increased circulating alanine further supports enhanced gluconeogenic activity through the glucose-alanine cycle. Moreover, substrate preference for gluconeogenesis varies with age: younger PCOS mice rely primarily on fatty acids, whereas older mice increasingly depend on amino acids. This shift toward amino acid-driven gluconeogenesis suggests progressive hepatic metabolic inflexibility, an indicator of IR that may help explain the declining metabolic adaptability observed with ageing in PCOS. Androgen excess provides an additional layer of hepatic IR. Elevated DHT directly activates hepatic ARs, triggering intracellular signaling events that inhibit insulin action. Activated ARs interact with the p85 subunit of PI3K, limiting its activation and reducing AKT phosphorylation, thereby allowing FOXO1 to remain active and sustain expression of *Pck1* and *G6pc* (**Figure 5**), two key gluconeogenic enzyme genes (51). This androgen-dependent amplification of gluconeogenesis creates a persistent cell-autonomous IR within the liver. Recovery of insulin signaling and normalization of gluconeogenic output in liver-specific AR-knockout models underscore that hepatic AR signaling is both necessary and sufficient to drive this phenotype (51). In addition to receptor-mediated effects, enzymatic alterations further exacerbate hepatic metabolic dysfunction in PCOS rats. Reduced glucokinase expression, together with increased hexokinase levels, shifts hepatic glucose phosphorylation capacity toward low-volume, high-affinity hexokinase activity. Unlike glucokinase, hexokinase cannot effectively manage postprandial glucose loads, making this shift a maladaptive attempt to preserve glucose phosphorylation despite impaired insulin signaling. This imbalance contributes to inefficient hepatic glucose utilization and increases reliance on gluconeogenesis (52).

### 3.6 Changes in endometrial tissue

Endometrial energy and glucose regulation in PCOS is a multifactorial disorder of insulin signaling involving inflammation, glucocorticoid imbalance, cytoskeletal dysfunction, and androgen-induced metabolic reprogramming. The endometrium of patients with PCOS exhibits increased expression of *INSR* and *IRS1/IRS2* mRNA, with both insulin and androgen exposure further enhancing IRS tyrosine phosphorylation (53). Although these changes suggest a compensatory attempt to maintain insulin sensitivity, they do not translate into improved downstream signaling, indicating that early insulin receptor proximal sensitization is reduced by inhibitory mechanisms operating further along the pathway. Local inflammation is a noticeable inhibitory factor within endometrial tissue. Elevated tumor necrosis factor-alpha (TNF-alpha) levels and increased phosphorylation of NFkappaB p65 induce inhibitory serine phosphorylation of IRS1, which diminishes its ability to transmit insulin signaling and simultaneously reduces GLUT4 expression (54). These inflammatory changes resemble patterns observed in other insulin-resistant tissues but appear to be particularly prominent in endometrial stromal cells of women with PCOS, especially in the presence of obesity. A second major disruptor of endometrial insulin action is altered intracrine glucocorticoid metabolism. Reduced local expression of 11beta-HSD2 leads to excessive accumulation of cortisol relative to cortisone, markedly increasing the local cortisol-to-cortisone ratio. Elevated cortisol further strengthens IR by inducing inhibitory IRS1 phosphorylation, reducing AKT activation, and increasing PTEN (55). These findings indicate that glucocorticoid imbalance may amplify inflammation-driven suppression of insulin signaling, creating a dual inhibitory environment that overrides the otherwise increased expression of insulin signaling components. Alterations in the endometrial cytoskeleton also contribute to disrupted glucose regulation. Decreased TALIN-1 expression weakens the integrin-actin connection, which is essential for proper GLUT4 translocation within the cell. Reduced TALIN-1 also correlates with diminished GLUT4 levels and impaired insulin-stimulated glucose uptake in both the endometrial tissue of patients with PCOS and IR and the PCOS mouse model (56). Because integrin-mediated mechanotransduction supports cellular responsiveness to metabolic signals, the loss of TALIN-1 suggests that impaired cytoskeletal signaling may represent a structural limitation on insulin responsiveness in the endometrium. In addition to these inhibitory processes, androgen excess changes endometrial glucose metabolism toward insulin-independent pathways. Exposure to DHT increases expression of GLUT1 and GLUT12 in human endometrial stromal cells and remodels metabolic gene networks toward glycolysis, gluconeogenesis, and carbohydrate-processing pathways, while downregulating transcripts involved in regulated glucose transport and glucokinase-related metabolic control (53).

### 3.7 Systemic changes

#### 3.7.1 Systemic insulin signaling and glucose transport disruption

In the DHT-induced PCOS mouse model, DHT induced tissue-specific changes in insulin signaling and glucose transport, thereby separating energy storage (liver and WAT) from reproductive tissues. Hepatic insulin signaling was compromised: *Insr, Irs1*, and *Pi3kcd* mRNA levels were downregulated, and GLUT1/GLUT2 protein expression was decreased, with no changes in INSR, IRS1, or IRS2. Functionally, AKT phosphorylation was impaired (blunted insulin-stimulated p-AKT and reduced basal p-AKT in the fed state), with downstream effects including reduced basal and insulin-stimulated glucose uptake and decreased plasma membrane GLUT1. Similar effects could be seen in WAT, with the difference being reduced *Ir* and *Irs1* mRNAs, and lowered GLUT1/GLUT4 levels. Both the pituitary and ovaries showed elevated GLUT1 and unchanged basal and insulin-stimulated glucose uptake, despite higher plasma-membrane GLUT1 levels than in controls. In the pituitary, insulin signaling was enhanced at the transcriptional level with higher *Ir* and *Irs1/2* and at the transporter level with increased GLUT1. GLUT2 and IRS1/2 levels did not change in either tissue. They also diverged in signaling pituitary insulin-evoked p-AKT was enhanced, while ovarian p-AKT remained at control levels. The latter also uniquely displayed a twofold increase in *Ir, Irs1*, and *Pi3kcd* mRNA and higher INSRbeta-subunit, changes not observed in the pituitary (45). Similar effects were also observed in letrozole-treated mice.

#### 3.7.2 Inflammation and immune-mediated mechanisms

**Figure 6:**
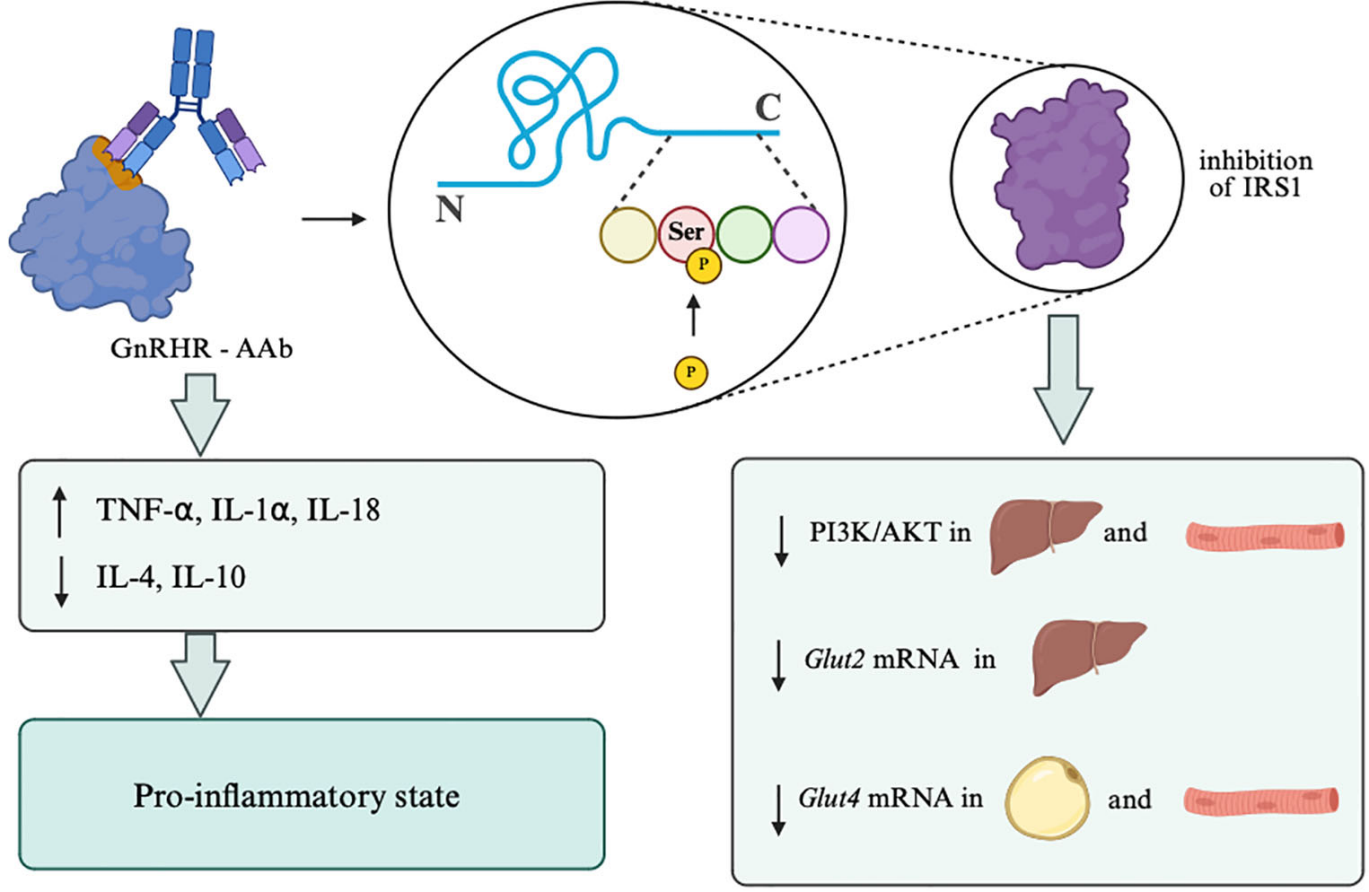
Proposed immune-mediated systemic mechanism contributing to insulin resistance in PCOS. GnRHR-activating autoantibodies promote inhibitory serine phosphorylation of IRS1, thereby attenuating PI3K/AKT signaling in liver and skeletal muscle. This is accompanied by reduced *Glut2* expression in hepatic tissue, reduced *Glut4* expression in white adipose tissue and skeletal muscle, and a cytokine shift toward a pro-inflammatory state characterized by increased TNF-alpha, IL-1alpha, and IL-18 and reduced IL-4 and IL-10. Created in BioRender. Sas, O. (2026) https://BioRender.com/98bpbj0.

The C-C motif chemokine receptor 5/C-C motif chemokine ligand 5 (CCR5/CCL5) chemokine axis is a pro-inflammatory pathway that is activated alongside impaired insulin signaling in VAT. PCOS mice exhibit increased expression of proteins involved in this pathway. Compared to controls, mRNA levels of *Ccr5* and *Ccl5* were elevated in perigonadal WAT; similarly, *Ccl5* mRNA was also increased in the ovary. In contrast, *Ccr5* mRNA expression was reduced in liver and skeletal muscle, with no corresponding increase in *Ccl5* in those tissues, suggesting a tissue-specific upregulation concentrated in VAT (and partly in the ovary). Consistent with IR observed at the signaling level in perigonadal WAT, decreased AKT phosphorylation and increased inhibitory serine phosphorylation of IRS1 at Ser307 in letrozole-treated mice were also observed. However, IRS1 tyrosine phosphorylation at Tyr941 remained largely unchanged, indicating a post-receptor impairment in the IRS1/AKT pathway. Correlation analyses linked higher CCR5 expression with higher testosterone, body weight, fasting insulin, and HOMA-IR (but not fasting glucose), whereas CCL5 did not correlate with testosterone but did correlate with body weight, fasting glucose, fasting insulin, and HOMA-IR. It is indicated that upregulated CCR5/CCL5 signaling in VAT is associated with worsened metabolic IR in this PCOS model, though the evidence is associative rather than a direct causal demonstration (24). New evidence sheds light on autoantibody-associated insulin impairment in PCOS: in many patients, an activating autoantibody directed toward the second extracellular loop of the gonadotropin-releasing hormone receptor (GnRHR) is present and indicative of IR. In a female rat model, immunization-induced GnRH receptor-activating autoantibodies (GnRHR-Aab) inhibited IRS1 via inhibitory serine phosphorylation at S636/639. This, in turn, further attenuated PI3K/AKT signaling in both liver and skeletal muscles. Reflecting the same mechanistic pathway, *Glut* mRNA expression was decreased – *Glut2* in hepatic tissue and *Glut4* in WAT and skeletal muscle. This is further supported by the observed shift in cytokine profile toward a pro-inflammatory state, characterized by higher levels of TNF-alpha, IL-1alpha, and IL-18, and lower levels of IL-4 and IL-10 in rats with GnRHR-Aab (**Figure 6**) (57).

#### 3.7.3 Chronobiological disruption

PCOS-related IR reflects not only the intensity of insulin signaling but also its chronobiological timing. PCOS-like rats display disrupted hepatic clock function, with reduced brain and muscle arnt-like protein 1 (BMAL1) (a key transcription factor that regulates the NAMPT/NAD+/SIRT1 pathway) and elevated cryptochrome circadian regulator 2 (CRY2) levels in a time-dependent manner. This, together with decreased p-AKT protein levels, implies an association between circadian clock genes and IR. Disturbances in SIRT1-NAMPT rhythms and altered timing of peroxisome proliferator-activated receptor gamma (PPARG) and GLUT4 point to hepatic IR caused by activating metabolic pathways at the wrong time of day. The adipose tissue of these rats shows a similar time-dependent dysregulation, with decreased BMAL1 protein and increased circadian locomotor output cycles kaput (CLOCK) protein at inappropriate circadian time points. Despite no change in *Glut4* mRNA over time, reduced p-AKT levels imply defective glucose signaling rather than transcriptional loss. In these cells, testosterone treatment reduces BMAL1 and disrupts the NAMPT/NAD+/SIRT1/GLUT4 pathway, leading to IR. However, *Clock*, *Per1*, and *Per2* stability in a hyperandrogenic state shows a BMAL-1-focused defect rather than systemic clock impairment (58). Additionally, prolonged exposure to darkness disrupts hepatic circadian clocks (more than adipose) and reduces *Bmal1, Clock*, and *Per1/Per2* mRNA expression. This coincides with impaired insulin signaling in liver tissue, as evidenced by reduced p-AKT and lower *Glut4*, with no difference in *Insr* mRNA. Both constant darkness and constant light rats exhibited lower hepatic *Ar* mRNA expression; however, AR protein expression was increased only in the prolonged darkness group. While broadly similar to hepatic findings, adipose tissue from rats kept in darkness showed lower *Insr* mRNA levels but maintained p-AKT activity. Apart from changes in circadian clock genes, prolonged darkness lowers hepatic sex hormone-binding globulin *(Shbg)* and insulin-like growth factor binding protein 4 (*Igfbp4)* transcripts, as well as SHBG. *In vitro* studies confirm that *PER1/2* loss in HepG2 cells reduces SHBG and IGFBP4 expression, implicating hepatic PER1/2 as positive regulators that limit androgen bioactivity (59).

#### 3.7.4 Alterations related to gut microbiota

Emerging evidence implicates the gut microbiota in the pathogenesis of IR in PCOS. Women with PCOS carry more genes related to IR and insulin signaling, as well as to several inflammatory signaling pathways (mTOR, PI3K/AKT, JAK/STAT, MAPK), compared to women without PCOS. Serum metabolomic profiling identified 35 metabolites that differed between PCOS and controls. In particular, the ceramide species Cer (d16:2/22:0) and the fatty acid 3Z,6Z,9Z-pentacosatriene correlated positively with fasting glucose, insulin, and HOMA-IR. These associations were functionally validated by fecal microbiota transplantation from donors with PCOS into pseudo-sterile rats, which led to fasting hyperglycemia, hyperinsulinemia, and higher HOMA-IR. However, transplantation of microbiota from healthy donors to letrozole-induced PCOS rats reversed these abnormalities (60). Contrary to the above, a study by Yang et al. did not observe any difference in a PCOS mouse model receiving fecal microbiota transplantation from either healthy donors or donors with PCOS. Additionally, healthy mice receiving fecal microbiota transplantation from individuals with PCOS developed IR but showed no abnormalities in glucose metabolism. Antibiotic treatment improved insulin function and increased farnesoid X receptor (*FXR*) mRNA levels in the ileum. Treatment with chenodeoxycholic acid (CDCA), an FXR agonist, improved both fasting and mean blood glucose levels. Both patients with PCOS and PCOS-like mice show a high abundance of Bacteroidetes, which can hydrolyze CDCA through microbial bile salt hydrolases. It is probable that, in patients with PCOS and in model mice, CDCA metabolism by Bacteroides is enhanced, leading to downregulation of activated intestinal FXR. The authors conclude that the metabolic disorders in PCOS may partly stem from insufficient activation of intestinal FXR (61).

#### 3.7.5 Pancreatic beta cell dysfunction

**Figure 7:**
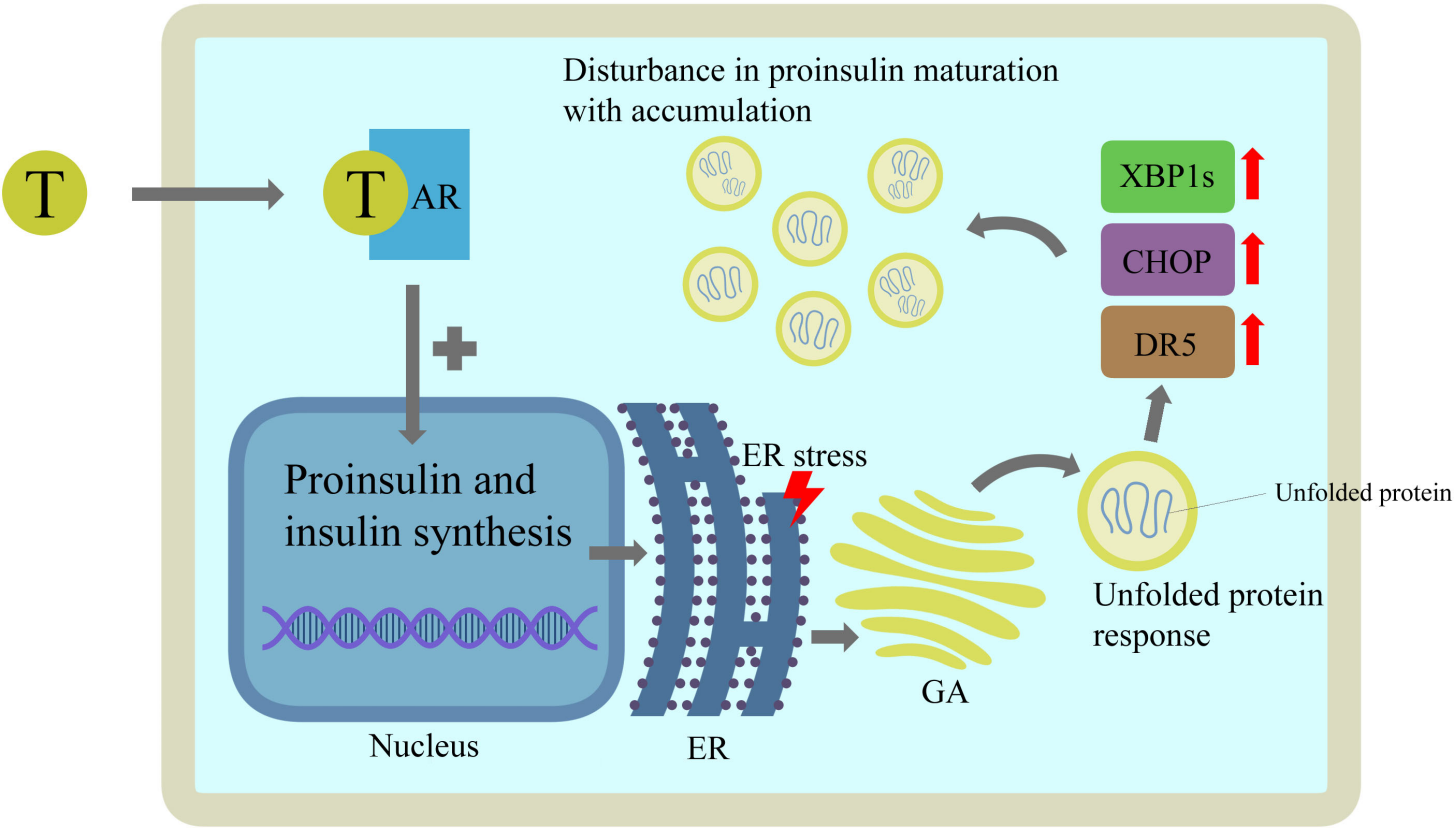
Chronic androgen exposure promotes endoplasmic reticulum stress and defective proinsulin maturation in pancreatic beta cells in PCOS. Testosterone acts through the AR to increase proinsulin and insulin synthesis, overloading ER protein-folding capacity. This induces ER stress and activates the unfolded protein response, leading to upregulation of spliced XBP1s, CHOP, and DR5, and to impaired proinsulin maturation and accumulation within beta cells.

Pancreatic beta cells experience testosterone-induced endoplasmic reticulum (ER) stress and defective proinsulin maturation, thereby intensifying hyperglycemia. Chronic androgen exposure in the islets of a mouse PCOS model, acting via AR, increased proinsulin and insulin synthesis, thereby overloading the ER protein-folding capacity. ER stress triggers the unfolded protein response system with upregulation of proapoptotic spliced X-box binding protein 1 (XBP1), C/EBP homologous protein (CHOP), and death receptor 5 (DR5). Functionally, this manifests as a disturbance in proinsulin maturation, as evidenced by an increased serum proinsulin-to-insulin ratio and disproportionate accumulation of proinsulin relative to insulin in isolated islets (**Figure 7**). Crucially, treatment with flutamide partially reversed these changes, indicating that androgen signaling-driven ER stress is a mechanistic source of both increased insulin secretion and impaired peptide processing in beta cells in PCOS (62).

#### 3.7.6 miRNA-mediated dysregulation

Among women with PCOS compared to controls, 46 miRNAs show significant expression changes. miR-486-5p, miR-223-3p, miR-6088, and miR-122-5p are differentially expressed, with miR-122-5p being the most strongly associated with IR in the PCOS cohort. The downstream targets of these miRNAs are associated with pathways involving insulin signaling, cellular protein distribution, developmental processes, and various metabolic and inflammatory pathways. More specifically, miR-122-5p targets genes involved in the negative regulation of GTPase activity, tight junction organization, protein localization, and epithelial morphogenesis. At the cellular level, these genes target chloride channels and endosomal sorting complex required for transport (ESCRT) and ESCRT1. Pathway analysis showed enrichment in the tricarboxylic acid cycle and mucin-type O-glycan biosynthesis, and network analysis identified glucose-6-phosphatase subunit 3, aldolase A, and chloride intracellular channel 4 as interacting agents. Overall, the data point to miR-122-5p as an important regulator of mitochondrial metabolism, glycosylation, and membrane trafficking underlying IR in PCOS (63).

## 4 Discussion

This scoping review explored dysregulation in molecular carbohydrate metabolism and insulin signaling in PCOS across metabolic tissues (skeletal muscle, adipose tissue, and liver), the endometrium, and ovarian tissue. The included evidence comprised observational human studies with direct tissue sampling, ex vivo tissue analyses, animal models, and *in vitro* experiments, with notable methodological heterogeneity but overall consistent pathophysiological findings. Taken together, these observations support the view that PCOS represents an integrated metabolic and reproductive disorder, in which systemic IR, hyperandrogenism, and localized molecular perturbations combine and promote dysregulated glucose metabolism.

Four principal mechanistic themes emerged across studies. First, post-receptor defects in INSR/IRS signaling, with impaired downstream PI3K/AKT activation and GLUT4 expression and trafficking, were observed in skeletal muscle, adipose tissue, and in GCs or ovarian models (9, 15, 27, 43, 44, 46). Second, PCOS metabolic dysfunction was frequently framed as hepatic gluconeogenic drive and metabolic inflexibility plus skeletal muscle mitochondrial impairment, and these systemic phenotypes may be influenced by gut–liver axis alterations (41, 48–52, 60, 61). Third, chronic low-grade inflammation and adverse adipokine signaling, including chemerin, SAA1, and CCL5-linked effects, recurrently converged on inhibitory IRS/AKT signaling and reduced glucose transport (15, 19, 24, 48, 49). Finally, these pathways appeared to be further modulated by androgen excess, obesity, and epigenetic regulation (m^6^A, miRNA, and circadian disruption) (22, 23, 43, 45, 51, 59, 63).

One possible explanation for why different mechanisms predominate across tissues is that androgen excess disrupts the physiological function of each tissue. In metabolic tissues such as the liver and adipose tissue, DHT primarily drives defects in insulin-mediated glucose handling, reduced PI3K/AKT signaling, reduced glucose uptake, and, in the liver, increased gluconeogenic drive because these tissues are specialized for systemic glucose homeostasis (45, 64). By contrast, in reproductive tissues, insulin and androgen signaling normally act as co-regulators of gonadotropin responsiveness, follicular growth, steroidogenesis, and endometrial differentiation; accordingly, androgen excess is more likely to manifest there as altered ovarian and endometrial signaling than as a classic hepatic-type gluconeogenic phenotype (53, 65, 66).

Our findings are similar to several recent reviews that have summarized IR in PCOS, either by focusing on mechanisms in tissues targeted by insulin or by providing broader overviews of pathogenesis and clinical management (67–69). These reviews consistently indicate that post-receptor defects in insulin signaling, chronic inflammation, and androgen excess interact and exacerbate metabolic and reproductive function. However, the available data often focus on a single organ system or on clinical consequences rather than systematic mapping (70–72). Our scoping review extends this literature by combining molecular data from skeletal muscle, adipose tissue, liver, ovarian tissue, and endometrium, explicitly focusing on carbohydrate metabolism and by mapping recurring mechanistic themes and evidence gaps rather than evaluating intervention effects or isolated pathways. This map may help clinicians and researchers understand the molecular mechanisms of metabolic risk in PCOS and to identify directions for future work, especially in connection with PCOS guidelines, which highlight IR and cardiometabolic risk as key clinical concerns (3, 4, 73).

A deeper understanding of the molecular basis of PCOS can lead to the development of novel treatment strategies that improve the lives of PCOS patients. Lifestyle changes, such as modifying diet (74, 75) and increasing physical activity (43, 76),, should be considered first-line treatments for managing patients with PCOS and obesity. However, when these methods prove to be ineffective, pharmacological treatment is considered. Currently, metformin remains the second most popular drug in the pharmacological management of PCOS, just after combined oral contraceptives, mainly due to its broad effects on cellular signaling pathways linked to IR in PCOS (77). Studies show that GLP-1 receptor agonists (78) or SGLT2 inhibitors (79) could be novel drug candidates for the treatment of PCOS. Studies also reveal that combinations of different drugs yield greater benefits than using a single drug alone (80, 81). It should be kept in mind that the existing therapeutic approaches mostly focus on relieving symptoms and metabolic side effects of PCOS rather than eliminating the disorder. **Table 1** lists the possible interventions available for managing PCOS and the molecular pathways involved.

**Table 1:** Dietary and pharmacological interventions targeting molecular pathways involved in insulin resistance in PCOS.

Pathway/axis	Key mechanism	Dietary or pharmacological treatment
INSR/IRS–PI3K/AKT–GLUT4	altered INSR/IRS regulation, inhibitory IRS serine phosphorylation, reduced PI3K/AKT activation, impaired GLUT4 expression	Metformin (82), melatonin (83)
TLR2/4–NF-κB/chemerin	TLR2/4–NF-κB, chemerin-CMKLR1, and related inflammatory inhibition of IRS/AKT signaling and glucose transport	Metformin (84, 85), resveratrol - needs further research (86)
CRP–IRS/AKT	liver-focused inflammatory pathway linking CRP and NETs with impaired INSR/AKT, reduced glycolysis, lower GLUT2/4, and increased gluconeogenic drive	Metformin (87), hypocaloric diet (88), fortified yogurt (89)
MAPK/ERK–JNK/p38	altered MAPK/ERK signaling and stress-kinase activation via JNK/p38, contributing to IR	Metformin (90), pioglitazone (91)
AMPK–mTORC1–TGFβ/SMAD	metabolic remodeling pathway involving AMPK, mTOR/mTORC1, TGFβ-SMAD; particularly relevant to skeletal muscle in IR, fibrosis/ECM remodeling and altered GLUT4-related responses	Myo-inositol (92), metformin (93)
AR–PI3K/AKT	AR supresses PI3K/AKT and increases Pck1/G6pc expression, promoting gluconeogenesis and IR in liver	Metformin (93, 94)

### 4.1 Limitations

Several limitations of this review should be acknowledged. First, the search was restricted to selected peer-reviewed articles published in English, and grey literature was not systematically searched. As a result, publication bias cannot be excluded, and studies reporting positive or more novel mechanistic findings may have been more likely to be published and therefore captured by this review. Second, the underlying evidence is highly heterogeneous across experimental models and measures used to assess IR and carbohydrate metabolism. This heterogeneity introduces a risk of misclassification of tissues, pathways, or outcomes. Third, no formal critical risk-of-bias assessment or grading of certainty for individual studies was performed; therefore, this mechanistic molecular map should not be regarded as a definitive evaluation of causal mechanisms. In addition, a substantial proportion of the mechanistic evidence identified in this review derives from rodent and *in vitro* models. Although these studies are valuable for identifying candidate pathways and establishing mechanistic plausibility, findings supported only by such models should be interpreted cautiously and should not be assumed to directly reflect human PCOS biology. Their translation is limited by several factors, including differences specific to certain species in ovarian physiology and systemic metabolism; differences between experimentally induced PCOS-like states and the heterogeneous clinical phenotype of human PCOS; and the artificial nature of *in vitro* conditions, which do not fully recreate the *in vivo* milieu. Finally, the available literature is uneven across tissues and among populations representing specific PCOS phenotypes; moreover, many studies are relatively small, cross-sectional, and rely on indirect measures of IR. In particular, HOMA-IR, which was used in a substantial proportion of the included studies, primarily reflects fasting hepatic IR and is less informative for peripheral insulin sensitivity. Therefore, associations between HOMA-IR and tissue-specific molecular findings should be interpreted with caution. Moreover, in cross-sectional studies, it is difficult to determine whether the molecular alterations observed are causal, secondary to PCOS, or adaptive responses to hyperinsulinemia, hyperandrogenism, obesity, or other metabolic abnormalities. In addition, most studies were conducted in China, with limited data from other regions, which may restrict the generalizability of these findings to broader populations.

### 4.2 Conclusions

The synthesis of molecular data from metabolic (skeletal muscle, adipose tissue, liver) and reproductive (GCs, endometrium) tissues indicates that PCOS is characterized by disturbances in insulin signaling and glucose metabolism, with substantial tissue- and model-specific heterogeneity. Across tissues, the most consistent pattern involves post-receptor insulin signaling impairment (altered INSR/IRS regulation, inhibitory IRS serine phosphorylation, reduced PI3K/AKT activation, and impaired GLUT4 expression and trafficking), often accompanied by inflammatory signaling that converges on the same pathway nodes (9, 15, 19, 43, 44, 48, 49, 54–56). In parallel, several studies highlight mitochondrial and glycolytic dysfunction (particularly in GCs and skeletal muscle), supporting an energy-metabolism component of IR (28–30, 41, 42). Findings in liver tissue particularly emphasize a shift toward a gluconeogenic, insulin-resistant phenotype driven by inflammatory mediators and androgen receptor-dependent suppression of PI3K/AKT signaling (48–52). Systemic-level modifiers, including hyperandrogenism, obesity, epigenetic and non-coding RNA regulation, circadian disruption, environmental endocrine disruptors, autoantibody-associated inflammatory mechanisms, gut microbiome, and FXR-related pathways (with some conflicting results), and beta cell ER stress affecting proinsulin processing, may shape the onset, tissue distribution, and severity of metabolic dysfunction in PCOS (22, 23, 39, 40, 45, 51, 57–63).

## Data Availability

The original contributions presented in the study are included in the article/**Supplementary Material**. Further inquiries can be directed to the corresponding author.
