# Supplementary material for: Molecular mechanisms of insulin resistance and altered carbohydrate metabolism in PCOS: a scoping review
Source: Front Endocrinol (Lausanne). 2026 Apr 13;17:1810805. doi: 10.3389/fendo.2026.1810805 (PMC13111087; doi:10.3389/fendo.2026.1810805)
Supplement: Supplementary file 2 [file Table2.docx]

**Supplementary table S2. Characteristics of included studies.** List of abbreviations: NR - not reported, H - human model study, A - animal model study, H/A - human and animal model study, Ad-CT - adenoviral control vector, Ad-sirklotho - adenoviral vector expressing siRNA against Klotho, AhR/AHR - aryl hydrocarbon receptor, AKT - protein kinase B, AKT1 - AKT serine/threonine kinase 1, AML12 - AML12 mouse hepatocyte cell line, AMPK - AMP-activated protein kinase, ANGPTL2 - angiopoietin-like 2, AR - androgen receptor, AS160 - Akt substrate of 160 kDa, ATF4 - activating transcription factor 4, BMAL1 - brain and muscle ARNT-like 1, BPA - bisphenol A, CCR5 - C-C motif chemokine receptor 5, CCL5 - C-C motif chemokine ligand 5, CMKLR1 - chemokine-like receptor 1, CRP - C-reactive protein, DHEA - dehydroepiandrosterone, DHEAS - dehydroepiandrosterone sulfate, DHT - dihydrotestosterone, DNase I - deoxyribonuclease I, ECL2 - second extracellular loop, ECM - extracellular matrix, ER - endoplasmic reticulum, ERK - extracellular signal-regulated kinase, FMT - fecal microbiota transplantation, FOXO3 - forkhead box O3, FSH - follicle-stimulating hormone, FSHR - follicle-stimulating hormone receptor, GCs - granulosa cells, GIR - glucose infusion rate, GK - glucokinase, GLUT1 - glucose transporter type 1, GLUT4 - glucose transporter type 4, GM3 - ganglioside GM3, GPR1 - G protein–coupled receptor 1, GSH - glutathione, hCG/HCG - human chorionic gonadotropin, HEK293 - human embryonic kidney 293 cells, HK1 - hexokinase 1, HKDC1 - hexokinase domain-containing protein 1, HMGB1 - high mobility group box 1, HOMA-IR - Homeostasis Model Assessment of Insulin Resistance, IGFBP4 - insulin-like growth factor binding protein 4, IGFIR - insulin-like growth factor 1 receptor, IL - interleukin, IR - insulin resistance, IRS2 - insulin receptor substrate 2, IVF - in vitro fertilization, JNK - c-Jun N-terminal kinase, L. reuteri - Lactobacillus reuteri, LHR - luteinizing hormone receptor, MAPK - mitogen-activated protein kinase, MIF - macrophage migration inhibitory factor, mTOR - mechanistic target of rapamycin, mTORC1 - mechanistic target of rapamycin complex 1, mTORC2 - mechanistic target of rapamycin complex 2, NETs - neutrophil extracellular traps, pAKT - phosphorylated AKT, PBS - phosphate-buffered saline, PEPCK - phosphoenolpyruvate carboxykinase, PER1/2 - period circadian regulator 1/2, PFKM - phosphofructokinase (muscle), PFKP - phosphofructokinase (platelet), PGK1 - phosphoglycerate kinase 1, PHLPP1 - PH domain leucine-rich repeat protein phosphatase 1, PI3K - phosphoinositide 3-kinase, PKM - pyruvate kinase (muscle), PTEN - phosphatase and tensin homolog, RU486 - mifepristone, SAA1 - serum amyloid A1, SAM68 - Src-associated in mitosis, 68 kDa, SHBG - sex hormone-binding globulin, siRNA - small interfering RNA, si-NC - negative control siRNA, SKP2 - S-phase kinase–associated protein 2, TCA - tricarboxylic acid, TGFbeta - transforming growth factor beta, UPR - unfolded protein response, WAT - white adipose tissue

| Author, publication year | Study model and design | Country | Sample group data | Tissue/material  for molecular  investigation | Limitations of the study,  future research directions |
| --- | --- | --- | --- | --- | --- |
| Andrisse et al., 2018 | A | USA | Human: NR | Hepatic and  pituitary cells | The number of animal subjects was not stated. Targeting the tissue-specific consequences of androgen excess - promising direction for future PCOS therapies. Deeper exploration of glucose metabolism and insulin pathways in skeletal muscle is necessary to better understand its role in the metabolic profile of PCOS. |
|  | Animal: in vivo, ex vivo, and in vitro models |  | Animal: CD1/129SvJ/C57BL6 mice: number: NR; PCOS induction: DHT |  |  |
| Andrisse et al., 2021 | H/A | USA | Human: 3 | Hepatic tissue | The number of animal subjects was not stated. Future studies should investigate additional molecular pathways which could be associated with hyperandrogenemia-induced impairment of hepatic insulin signaling. The authors do not exclude the possibility that these pathways could involve mitochondrial dysfunction. |
|  | Human: in vitro study; Animal: in vivo, ex vivo and in vitro studies |  | Animal: C57BL/6 mice; number NR; PCOS induction: DHT |  |  |
| Belani et al., 2018 | H | India,  Canada | Human: 39 PCOS, 30 controls | Granulosa cells | NR |
|  | Human:  case-control and ex vivo study |  | Animal: NR |  |  |
| Chahal et al., 2021 | H/A | India, USA, France | Human: 61 PCOS,  41 controls | Granulosa cells, HEK293 cells expressing FSHR and LHR | The study doesn't fully explore the specific tyrosine phosphorylation sites in IRS2 in response to FSH.The study doesn't assess the long-term effects of FSH and LH on glucose metabolism in granulosa cells.More clinical studies are required to validate the findings in PCOS patients and explore therapeutic potential of LH antagonists. |
|  | Human: case-control and in vitro models; Animal: in vivo and in vitro study |  | Animal: Holtzman strain rats; 45 (35 FSH/hCG + 10 controls with saline); PCOS adult rat model: 18 (12 controls + 6 RU486); Additional rats used for in vitro GCs isolation and molecular studies but exact group sizes are not detailed; PCOS induction: RU486 |  |  |
| Chen et al., 2022 | H/A | China | Human: 50 PCOS, 50 controls | Granulosa tumor cell line | Not fully clarified FOXO3’s role with insulin stimulation. Additional confounding factors in human PCOS/IR were not fully addressed. Larger or more refined cohorts and more extensive mechanistic analyses are needed. Further exploration of FOXO3’s activities with hyperinsulinemic conditions. More robust PCOS patient groups to confirm miR-29c-3p/FOXO3 pathways and its role. |
|  | Human: case-control, in vitro studies; Animal:  in vivo model;  In silico analysis |  | Animal: Sprague-Dawley rats; 120 induced PCOS rats and 20  normal (non-PCOS) controls,  PCOS induction: DHEA |  |  |
| Cong et al., 2024 | H/A | China | Human: NR | Ovarian granulosa-like tumor cell line, ovarian tissue | The number of animals used in the in vivo study was not explicitly stated. No direct human patient data. HKDC1’s role was tested in short-term experiments. The specific interplay with other hexokinase isoforms was not fully explored. Future directions: Confirm the clinical importance of HKDC1 in PCOS patients. Further research on interaction of HKDC1 with insulin resistance and chronic inflammation |
|  | Human:  in vitro study;  Animal:  in vivo experimental study;  In silico analysis |  | Animal: C57BL/6 mice; no data;  PCOS induction: DHEA |  |  |
| Di et al., 2018 | H | China | Human: NR | Granulosa cells, KGN cells | Further studies should examine ATF4's key role in dyslipidemia of PCOS. Aditionally, the precise mechanisms by which ATF4 affected the pathophysiological progress of PCOS requires further investigation. |
|  | Human: cross-sectional study with in vitro component |  | Animial: NR |  |  |
| Froment et al., 2022 | H/A | France | Human: 9 PCOS, 8 controls | Granulosa cells and KGN cells | There is a need to investigate the potential application of AMPK activators (e.g., metformin, adiponectin inducers) in the treatment of PCOS, particularly with the aim of personalizing treatment strategies based on AMPK activity levels in granulosa cells. Employing AMPK overexpression strategies in ovarian follicles of PCOS mouse models would help determine the therapeutic efficacy of upregulated AMPK signaling in reversing PCOS-related phenotypes. Development of AMPK-specific therapeutic targets could contribute significantly to addressing subfertility in PCOS, especially when associated with metabolic syndromes. |
|  | Human: case-control and in vitro models; Animal: in vivo study; In silico analysis |  | Animal: Wild-type male mouse, 2 female  alpha1AMPK-knock-out mice;  PCOS induction: NR |  |  |
| Gao et al., 2022 | A | China | Human: NR | Hepatic tissue | Firstly, the downstream evaluation of metabolomic results should be established. These include knocking-down genes or inhibiting enzymatic activities involved in the TCA cycle, gluconeogenesis, GSH metabolism, and protein digestion in the mouse liver with PCOS. Secondly, further studies investigating the effects of PCOS hepatic exosomes on the liver cells in vitro are warranted to verify the metabolic roles of exosomes in PCOS pathogenesis. Lastly, in order to comprehensively understand the metabolic status of liver tissue and hepatic exosomes in PCOS, multi-omics approaches should be integrated into the subsequent research. |
|  | Animal:  in vivo and ex vivo models;  In silico analysis |  | Animal: C57/BL6 mice; 5 young-age PCOS, 6 young-age normal, 8 middle-age PCOS, 5 middle-age normal; PCOS induction: DHT |  |  |
| Guo et al., 2018 | A | China | Human: NR | Serum, follicles | Number of animal subjects not stated. |
|  | Animal: in vivo and ex vivo experimental study |  | Animal: Wistar rats; number NR; PCOS induction: DHEA |  |  |
| Ha et al., 2021 | H | China | Human: PCOS group: 35 women with normal weight. Obesity + PCOS group: 35 women with PCOS and obesity.  Normal weight group: 35 women with normal weight. Obesity group: 35 women with obesity without PCOS. | Endometrial stromal cells | Endometrial tissue was collected in the proliferative phase and may not have well represented the microenvironment of peri-implantation |
|  | Human: cross-sectional, ex vivo and in vitro studies |  | Animal: NR |  |  |
| He et al., 2019 | H | China | Human: 67 PCOS, 63 controls | Granulosa cells | The study examined only a few core components and not the full components of the IGFIR/PI3K pathway. The use of luteinized granulosa cells, collected post-hCG trigger during IVF, may not accurately reflect early follicular signaling. This introduces potential bias in interpreting natural follicular development. Further studies are required to investigate function and regulation of IGFIR/PI3K pathway in human ovaries |
|  | Human: case-control study with ex vivo model |  | Animal: NR |  |  |
| Huang et al., 2021 | H | China | Human: 43 PCOS, 41 controls | Granulosa cells/ KGN cells | The association between CCNL expression and related endocrine parameters in the general population requires further investigation due to the limited availability of clinical data. Additionally, the authors were unable to asses CCNL expression and its impact on glucose uptake in the liver due to the scarcity of human hepatic tissue samples. It should be considered to examine the relationship between CCNL and protein expression in PCOS patients. Moreover, since most participants in this study were of Asian Han ethnicity, the findings should be cautiously applied to broader population. |
|  | Human: case-control, ex vivo and in vitro studies;  In silico analysis |  | Animal: NR |  |  |
| Huo et al., 2022 | H | China | Human: 76 PCOS, 30 controls | NR | The specific mechanism behind miR-122 and insulin resistance in PCOS needs further evaluation. To determine this it is necessary to collect more clinical specimens of patients with PCOS. There is a need to re-validate the results in ovarian tissue, further explore the effect of miR-122 on ovarian function in women with PCOS and further explore the role of miR-122 in animals and cell models. |
|  | Human: case-control study; In silico analysis |  | Animal: NR |  |  |
| Lee et al., 2020 | H | South  Korea | Human: 13 PCOS, 7 controls | Endometrial stromal cells/CRL-4003, endometrial cells from corpus of the uterus | There is a need to further explore the exact mechanism responsible for the increase in GLUT1 in PCOS. Additionally, it remains unclear how androgens, especially DHT, affect endometrial physiology. Further investigation is required to examine the differences in the molecular mechanisms of a hyperandrogenic environment in non-pregnant versus decidualized endometrium and its impact on glucose metabolism |
|  | Human: case-control study with in vitro component; In silico analysis |  | Animal: NR |  |  |
| Li et al., 2019 | H | China | Human: 26 controls without IR vs 24 controls with IR vs 25 PCOS without IR and 26 PCOS with IR | Granulosa-lutein cells | Further studies are needed to explore the relationship between lifestyle and chemerin levels in serum and follicular fluids from patients with PCOS. CMKLR1 and GPR1 receptors require further research. |
|  | Human: cross-sectional study with in vitro component |  | Animal: NR |  |  |
| Li et al., 2020 | A | USA | Human: NR | Hepatic tissue, skeletal muscle tissue, WAT, serum | Role of androgen excess in promoting inflamation in PCOS - still unclear. It will be of interest to evaluate the impact of IL-22 and other interleukins' administration in rat autoimmune model of PCOS. Further studies should focus on the potential importance of GnRHR-AAb in humans and in an animal model and to further evaluate inflamation and IR using this type of PCOS model. |
|  | Animal: in vivo, ex vivo and in vitro study |  | Animal: Sprague-Dawley rats; 8 PCOS, 8 controls; PCOS induction: (immunized) group were immunized with GnRHR ECL2 peptide and subsequently boosted with the same peptide in incomplete Freund's adjuvant. |  |  |
| Li et al., 2020 | H/A | China | Human: 20 PCOS, 20 control | HepG2 (hepatocytes), ovarian tissue, hepatic tissue, adipose tissue, serum | Inability to compare the expression of circadian clock genes in the liver or adipose tissue between women with and without PCOS, due to the limited availability of samples. Unable to determine the exact regulatory relationship between PER1 or PER2 and IGFBP4, SHBG, and AR in the liver. Unable to explore why the expression in the PAKT pathway remained unchanged under the same conditions. |
|  | Human: case-control study with in vitro component; Animal: in vivo experimental study |  | Animal: Sprague-Dawley rats; 90 (control, light, darkness), 56 ( control, darkness, darkness + rescue 1, darkness + rescue 2, darkness + xylooligosaccharides (XOS), darkness + Lactobacillus reuteri (L. reuteri), and darkness + melatonin); PCOS induction: NR |  |  |
| Li et al., 2021 | H/A | China | Human: 37 PCOS, 32 controls | Granulosa cells | Future studies should investigate the efficacy and safety of HSD11B1 inhibitors in PCOS treatment. Additionally, further research is needed to explore the various mechanisms involving HSD11B1 in PCOS pathophysiology. |
|  | Human: cross-sectional comparative study with in vitro component; Animal: in vivo study with ex vivo component |  | Animal: Sprague-Dawley rats; 24 (lentivirus infection), 20-21 per group (controls, PCOS, PCOS+BVT); PCOS induction: DHEA |  |  |
| Li et al., 2023 | A | China | Human: NR | Blood plasma | NR |
|  | Animal: in vivo and ex vivo study |  | Animal: rats; 150, PCOS induction: DHEA |  |  |
| Li et al., 2023 | H/A | China | Human: 15 PCOS , 15 controls | Ishikawa cells, endometrial cells | One of the limitations in this study was that they recruited endometrium from control patients, whereas women without any known pathological conditions may be more suitable. In addition, due to the limited number of cases included in this study, the correlation between PCOS phenotype and TALIN1 expression could not be analyzed. |
|  | Human: case-control and in vitro study; Animal: in vivo model |  | Animal: C57BL/6J mice; 12 Si-Talin1 on one side and si-NC on the other side, 6 si-Talin1 on both sides, 6 si-NC on both sides; PCOS induction letrozole |  |  |
| Lin et al., 2025 | H/A | China | Human: 40 PCOS, 19 controls (serum group), 17 PCOS, 19 controls (follicular fluid group) | AML12, NCTC1469 hepatic cells and bone marrow | NETs have more pronounced effect on AML12, than on NCTC1269 (different cellular characterisstic of these cell lines), inadequate sample size, incomlete information regarding the enrollment cohort (absence of postprandial blood glucose, insulin, lipid profiles, and inflammatory cytokine data), how PCOS triggers NET activation in liver was not studied. |
|  | Human: cross-sectional comparative study; Animal: in vivo and in vitro study; In silico analysis |  | Animal:  Sprague-Dawley rats; Control (n=9); PCOS (n=8; DHEAS); PDNaseI (n=9; DHEAS + DNase I); DNaseI (n=5; DNase I); PCOS induction: DHEAs |  |  |
| Liu et al., 2020 | H/A | China | Human: 44 PCOS (PCOS subdivided into 28 non-high-testosterone and 16 high-testosterone), 36 controls. | KGN and HEK293T cell lines | Clinical translation of PGK1’s role was not verified. Did not fully explore how other upstream or downstream regulators interact. Future directions: Investigation of additional metabolic pathways PCOS GCs. Validation of PGK1-AR-SKP2 axis in larger clinical populations. Evaluate potential therapies targeting PGK1 in combination with existing treatment. |
|  | Human: case-control and in vitro studies; Animal: in vivo models; In silico analysis |  | Animal: C57BL/6 mice; 3 groups (control, PCOS-like, and PCOS-like plus PTX) 8 mice each; PCOS induction: DHEA |  |  |
| Mao et al., 2018 | H/A | China | Human: 43 PCOS, 26 controls | Granulosa cells | This study does not investigate the level of KLOTHO protein in bloodstream in women with PCOS, the abortion rate and offspring’s survival rate in PCOS-induced rats. |
|  | Human: case-control study with in vitro component; Animal: in vivo and ex vivo studies |  | Animal: Sprague-Dawley rats; 30 PCOS, 30 control, 20 (10 PCOS Ad-CT virus injection, 10 PCOS Ad-sirklotho virus injection), 10 control (Ad-CT virus injection); PCOS induction: DHEA |  |  |
| Mazloomi et al., 2023 | H | Iran | Human: main group (45 PCOS, 45 controls) + validation group (15 PCOS, 15 controls) | Granulosa cells | There is a need to further investigate the epigenetic changes in glycolysis enzymes (HK1, PFKM, PFKP, and PKM) in granulosa cells. Further studies should also identify the factors responsible for low ATP levels in immature follicles of control women, which differ from those observed in women with PCOS. Additionally, it should be considered how the activity of key glycolytic enzymes, as well as other enzymatic and non-enzymatic cellular antioxidant agents, impacts granulosa cells in PCOS. Furthermore, the metabolic interactions between oocytes and their surrounding cells need to be established, as oocyte maturation is a complex process involving oocyte-cumulus complexes. |
|  | Human: case-control study with in vitro and ex vivo component |  | Animal: NR |  |  |
| Mićić et al., 2022 | A | Serbia | Human: NR | Subcutaneous adipose tissue, visceral adipose tissue | NR |
|  | Animal: in vivo and ex vivo study |  | Animal: Wistar rats:12 PCOS, 12 control; PCOS induction: DHT |  |  |
| Mićić et al., 2023 | A | Serbia | Human: NR | Skeletal muscle cells | Future studies should focus on the pathophysiology of PCOS in skeletal muscle, particularly in the context of obesity. Additionally, the authors suggest that AMPK activation in the endometrium should be investigated to better understand its role in PCOS. |
|  | Animal: in vivo and ex vivo study |  | Animal: Wistar rats: 12 PCOS, 12 control; PCOS induction: DHT |  |  |
| Mu et al., 2021 | A | China | Human: NR | Granulosa cells | Details of how miR-103 influences insulin resistance remain partly unclear, no direct human data, additional regulators in the PCOS were not fully explored. Further investigation of the mechanism by which miR-103 regulates insulin signaling, evaluating miR-103 inhibition as a therapeutic target in clinical settings. |
|  | Animal:  in vivo and in vitro models;  In silico analysis |  | Animal: Sprague Dawley rats; 10 control, 6 PCOS/non-IR, 54 PCOS/IR and 10 were used in preliminary procedures; PCOS induction: DHEA |  |  |
| Neisy et al., 2018 | A | Iran | Human: NR | Hepatocytes, uterine cells | NR |
|  | Animal: in vivo and ex vivo experimental study |  | Animal: Sprague-Dawley rats;  14 PCOS (4-non treatment, 5-quercetin,  6-ethanol), 21 control (1-non treatment, 2- quercetin, 3-ethanol); PCOS induction: DHEA |  |  |
| Qi et al., 2018 | H | China | Human:  Endometrial biopsy: non-PCOS (n = 18), PCOS without IR (n = 18), and PCOS with IR (n = 18) Endometrial epithelial cells: non-PCOS (n = 29) and PCOS (n = 21) | Endometrial  epithelial cells | The study may be limited by a small sample size, for example in some tests of statistical significance. The authors point out, that quantitative measurements of cortisol and its metabolic enzymes in the endometria of PCOS women have never been performed previously. The study concludes that the causes of diminished 11beta-HSD2 and the exact underlying molecular mechanisms involving PTEN should be further investigated, especially in non-PCOS insulin-resistant women. |
|  | Human: cross-sectional study with in vitro component |  | Animal: NR |  |  |
| Qin et al., 2021 | H/A | China | Human: 6 PCOS, 6 controls | Granulosa cells | The study’s sample sizes is too small and without direct protein measurements, miRNA effects on their targets remain speculative. Insulin resistance and inflammation in the mouse model weren’t quantified, and no miRNA knock‑out experiments were done to prove causality. Prenatal androgenization mouse model doesn’t fully replicate human PCOS, limiting real‑world relevance. Bigger cohorts are needed. |
|  | Human: case-control study; Animal: in vivo and in vitro studies; In silico analysis |  | Animal: ICR mice; 6 PCOS, 6 controls; PCOS induction: DHT |  |  |
| Seow et al., 2021 | Animal | Taiwan | Human: NR | Ovarian tissue, skeletal muscle tissue, hepatic tissue, perigonadal white adipose tissue | The mechanism of insulin resistance in PCOS, as well as the role of CCL5, is not yet fully understood. Further studies are needed to establish a direct link between CCR5, CCL5, and HOMA-IR in letrozole-induced PCOS mice. |
|  | Animal: in vivo study |  | Animal: C57BL/6 mice: 10 PCOS, 10 controls; PCOS induction: letrozole |  |  |
| Shen et al., 2019 | A | China | Human: NR | Skeletal muscle cells | The study noted the discrepancy between its findings in mice and results from human studies, which may be due to differences in skeletal muscle type and androgen levels. Additionally, the study focused on short-term effects, which might not reflect long-term metabolic changes. |
|  | Animal: in vivo and ex vivo study |  | Animal: C57BL/6 mice; 14 PCOS, 14 controls; PCOS induction: DHEA |  |  |
| Shen. et al., 2022 | H/A | China | Human: 10 PCOS IR, 10 PCOS non-IR, 10 controls | KGN cells | Other isoforms of SORBS1 were not assessed. Unclear role of CAP in the development of PCOS and associated insulin resistance. Unclear role of hyperactive Akt signaling in the development of PCOS. Number of animal subjects not stated. |
|  | Human: cross-sectional study with in vitro component; Animal: in vivo study;  In silico analysis |  | Animal: C57BL/6 mice; number NR; PCOS induction: DHEA |  |  |
| Shi et al., 2024 | H/A | China | Human: 136 PCOS, 120 controls | Granulosa cells | There are several potential endocrine targets for diagnosis and treatment of PCOS. However, the role and infuence of these endocrine indicators include GILU4 and AhR on PCOS classifcation and diagnosis need to be further clarifed in the future investigation. Number of animal subjects was not reported. More longitudinal studies and increased sample sizes, as well as a variety of human population, are needed to further deepen the association between BPA exposure and human health outcomes. |
|  | Human: case-control and in vitro models; Animal: in vivo study; In silico analysis |  | Animal: C57BL/6J mice; number: NR, PCOS induction: DHEA |  |  |
| Song et al., 2018 | A | China | Human: NR | C2C12 mouse myoblasts, skeletal muscle tissue, blood | The specific mechanisms by which testosterone induces IR in skeletal muscle have yet to be determined. Furthermore, it is uncertain whether mTOR signaling and mTORC1-regulated autophagy are altered in the skeletal muscle of DHEA-treated mice. |
|  | Animal: in vivo and in vitro models |  | Animal: C57BL/6 mice; 12 PCOS, 12 controls; PCOS induction: DHEA |  |  |
| Stepto et al., 2020 | H | Australia, UK | Human: 30 PCOS, 29 controls | Skeletal muscle tissue | Limitations: small sample size. Establishing role of hyperandrogenism in causing insulin resistance in PCOS via peripheral tissue insulin signaling and fibrosis. Additional human research (in vivo and in vitro), supported by appropriate animals studies, is warranted to elucidate the role of androgens, mTOR signalling, TGFbeta ligand signalling networks and ECM deposition in PCOS-specific insulin resistance. More research is needed to understand the PCOS-specific mTOR downregulation and its role in the intrinsic insulin resistance in skeletal muscle. The study could not distinguish between mTORC1 and mTORC2 signaling, warranting further research. |
|  | Human: case-control study with ex vivo component |  | Animal: NR |  |  |
| Tan et al., 2021 | H/A | China | Human: 41 PCOS,  41 controls | Granulosa cells | The molecular mechanism of upstream regulation of LNK is still not well understood. While LNK is shown to regulate FOXO3 function through the AKT pathway by influencing its phosphorylation and subcellular localization, the reason for FOXO3 upregulation in granulosa cells of PCOS patients is still unknown. Further research is needed to understand LNK's role in oocyte maturation and granulosa cell-oocyte interaction. Additionally, the study has not fully addressed hyperandrogenism, another key aspect of PCOS. |
|  | Human: case-control and in vitro models; Animal: in vivo study |  | Animal: C57BL/6 mice: 12 PCOS, 12 controls; PCOS induction: DHEA + fat-high diet |  |  |
| Vilariño-García et al., 2022 | H | Spain | Human: 25 PCOS,  25 controls | Granulosa cells | Mechanism whereby the tyrosine phosphorylation of SAM68 negatively regulates its RNA binding function is to be investigated in GCs. |
|  | Human: cross-sectional, ex vivo and in vitro study |  | Animal: NR |  |  |
| Wang et al., 2020 [[36](#_ENREF_36)] | A | China | Human: NR | Ovarian tissue | Unconfirmed mechanism behind ANGPTL2's role in PCOS, as well as other proteins' involved in its pathway. Future studies should establish the exact mechanisms by which metformin decreases ANGPLT2 levels and examine protein expression of ANGPTL2. Future studies should also provide mechanistic insights into ANGPTL2's role in IR and PCOS pathogenesis. |
|  | Animal: in vivo and ex vivo studies |  | Animal: Sprague-Dawley rats; 40 PCOS [20 PCOS, 20 PCOS + metformin], 20 controls; PCOS induction: letrozole |  |  |
| Yang et al., 2018 | H/A | China | Human: NR | Adipocytes, ovarian tissue | Limited focus on the broader systemic effects of miR-33b-5p and GLUT4 regulation. Lack of human clinical validation for the findings derived from rat models and in vitro studies. No direct human tissues. Small sample of rats. Need for further exploration of the  miR-33b-5p role in human PCOS  tissues. Clinical interventions targeting miR-33b-5p was not explored. |
|  | Human: in vitro study; Animal: in vivo study; |  | Animal: Sprague-Dawley rats; 4 groups 6 each (non-PCOS/non-IR, non-PCOS/IR, PCOS/non-IR, PCOS/IR), PCOS induction: insulin, HCG, high fat diet |  |  |
| Yang et al., 2021 | H/A | China | Human: 56 PCOS, 31 controls | Hepatic tissue, intestinal tissue | Adding bile acid supplementation and antibiotic treatments to the PCOS fecal transplantation experiment could have strengthened the findings. The study would have been enhanced by excluding vegetarians and those who had used probiotics, prebiotics or antibiotics before sample collection, though controlling participants' diets was challenging. Only one sample was collected per participant. |
|  | Human: cross-sectional study; Animal: in vivo study; In silico analysis |  | Animal: C57BL/6 mice; 8 PCOS, 8 controls; PCOS induction: letrozole |  |  |
| Yang et al., 2022 | H/A | China | Human: 32 PCOS, 18 controls | Serum, fecal samples | Small sample size and single-center design. Causality is primarily inferred by the FMT animal model. Authors highlight the need for larger multicenter investigations and longitudinal research. Future work should investigate specific microbial strains and mechanistic pathways including and potential clinical interventions targeting the gut microbiota. Further investigation is needed to determine whether gut micriobiota modulates ovarian function through the mediation of ganglioside GM3. |
|  | Human:  case-control study; Animal:  in vivo models;  In silico analysis |  | Animal: Sprague-Dawley rats; first FMT experiment (pseudo-sterile model):16 rats: 8 receiving fecal microbiota from PCOS donors (“PCOS” group) vs. 8 receiving sterile PBS (controls).  Second experiment (letrozole-induced PCOS): 24 rats, subdivided into 4 groups (6 rats/group): Control, Letrozole-only, Letrozole + Diane-35, Letrozole + FMT from healthy donors. PCOS modelling = letrozole groups (18 rats) vs. control group (6 rats); PCOS induction: Fecal microbiota transplantation (PCOS donor vs. control donor) in pseudo-sterile rats.  Letrozole-induced PCOS in another set of rats. |  |  |
| Yang et al., 2024 | H | China | Human: 30 PCOS, 15 controls | Granulosa cells/KGN cells | It remains to be established whether PHLPP1 also influences other glycolytic enzymes and signaling pathways. Notably, the current findings are based solely on in vitro experiments conducted on human ovarian granulosa KGN cells. Further in vivo research could provide more insightful and clinically relevant outcomes, particularly involving primary cultured granulosa cells from women with PCOS. |
|  | Human: in vitro, ex vivo and observational study |  | Animal: NR |  |  |
| Yang et al., 2024 | H/A | China | Human: 16 PCOS, 16 controls | KGN cells | The systemic *GRIM19* knockout (not granulosa cell-specific) resulted in altered glucose and lipid metabolism in these mice, thus not fully elucidating the cause of their changes in glucolipid metabolism. The granulosa cell-specific effect should be further studied. The role of RAC1's regulation of GLUT4 in granulosa cells' glucose metabolism is still unclear. Future studies are needed to confirm authors' hypothesis of GRIM19 deficiency on RAC1/GLUT4 glucose uptake pathway and the ERK pathway connection to the impaired ovulation in patients with PCOS. |
|  | Human: case-control and in vitro study; Animal: in vivo study; In silico analysis |  | Animal: C57BL/6J mice; 16 wild type (8 PCOS, 8 controls) + 16 GRIM19 +/- (8 PCOS, 8 controls); PCOS induction: DHEA + high-fat diet |  |  |
| Yang. et al., 2022 | H | China | Human: 11 obese PCOS, 12 non-obese PCOS | Granulosa cells | NR |
|  | Ηuman: cross sectional study with ex vivo and in vitro components;  In silico analysis |  | Animal: NR |  |  |
| Yi-fan et al., 2025 | H | China | Human: NR | KGN cells | Future studies should integrate phosphoproteomic profiling to map post-translational modifications, coupled with conditional knockout models to establish causal links between specific phthalate-protein interactions and PCOS phenotypic outcomes. |
|  | Human: in vitro study; In silico analysis |  | Animal: NR |  |  |
| Zhai et al., 2020 | A | China | Human: NR | c2C12 mouse myoblasts,  skeletal muscle tissue, blood | Authors did not detect the expression of circadian clock genes in the liver and adipose tissues of women with or without PCOS due to the scarcity of samples. Future research: Elaborating the role of BMAL1 in the contribution of hyperandrogenism to IR in PCOS.  The authors indicate the need for interventional studies using melatonin as well as more in-depth related molecular research. |
|  | Animal: in vivo and in vitro study |  | Animal: Sprague-Dawley rats: 30 PCOS, 30 controls; PCOS induction: DHEA |  |  |
| Zhang et al., 2020 | H | China | Human: 20 IR-PCOS, 20 NIR-PCOS, 25 controls | Luteinezed granulosa cells | Even though HMGB1 is the initiator of innate immune responses in cells other than granulosa cells, the specific mechanisms and pathways through which inflammation evokes insulin resistance in these cells remain unknown. Additionally, the origin of HMGB1 in follicular fluid is uncertain. It is speculated that HMGB1 affects both non-luteinized and luteinized human granulosa cells. However, this research has only been conducted on luteinized granulosa cells, highlighting the need to establish its effects on non-luteinized granulosa cells. In vivo studies should be conducted to explore the interactions between factors influencing insulin resistance in women with PCOS. An additional control group - women with insulin resistance but without PCOS - would add further value to the study. |
|  | Human: cross-sectional, ex vivo and in vitro studies |  | Animal: NR |  |  |
| Zhang et al., 2020 | Animal | China | Human: NR | Ovarian tissue | Small pilot design with only 20 rats. PCOS heterogeneity was not fully addressed. No direct functional assays were performed to confirm links between specific miRNAs and insulin resistance.  Future directions: further studies on miRNAs (e.g. miR-146, miR-30, miR-3585) and their role in insulin signaling. Larger cohorts and broader functional experiments to demonstrate epigenetic regulation of insulin resistance in PCOS. |
|  | Animal: in vivo study; in silico analysis |  | Animal:  Sprague-Dawley rat;  10 in the letrozole + high‑fat diet group, 10 controls;  PCOS induction letrozole + high‑fat diet |  |  |
| Zhou et al., 2018 | A | China | Human: NR | Ovarian tissue | Only a small animal sample. Regulatory details of MIF and MAPK interacting in PCOS remain unclear. No mechanistic inhibition experiments to confirm causality. Future direction: Further work to clarify how MIF is activated in PCOS, testing of MAPK or MIF inhibitors as a therapeutic option in PCOS, MIF may play a role as an early PCOS diagnosis biomarker. |
|  | Animal: in vivo and ex vivo study |  | Animal:  Sprague-Dawley rats; 10 controls, 20 PCOS (10 received normal diet and 10 received high-fat diet); PCOS induction with DHEA with or without high-fat diet |  |  |
| Zhu et al., 2021 | A | China | Human: NR | Insulinoma cell line | Rat cell lines were used to dissect the effect of androgens excess on ER stress and hyperinsulinemia, this study is unable to encompass the entire aspects of in vivo experiments, which need to be further addressed in future studies. |
|  | Animal: case-control in vivo study with ex vivo and in vitro components |  | Animal: C57BL/6 mice; 20-24 PCOS, 10-12 controls; PCOS induction: DHEA |  |  |
| Zhu et al., 2022 | H | China | Human: 22 controls without IR, 22 controls with IR, 22 PCOS without IR, 22 PCOS with IR | Granulosa cells | No limitations reported, authors stressed that the biological functions of SAA1 in the acute phase are still not fully understood. Examine if SAA1 can be used as biomarker for metabolic dysfunction in PCOS, confirm if SAA1 has relevence in insulin resistance on in vivo models |
|  | Human: cross-sectional study with in vitro component |  | Animal: NR |  |  |
| Zou et al., 2023 | A | China | Human: NR | Ovarian tissue | This study did not explore certain known factors that inhibit the PI3K/AKT pathway, such as Phosphatase and tensin homolog (PTEN) and c-Jun N-terminal kinase (JNK). Additionally, it did not confirm the hypothesis that the hypermethylation of SKIP mRNAs increases its expression, inhibiting the PI3K/AKT signaling pathway. |
|  | Animal: in vivo study; In silico analysis |  | Animal: C57BL/6 mice; 5 PCOS, 5 controls; PCOS induction: DHEA |  |  |
