# Supplementary material for: Molecular mechanisms of insulin resistance and altered carbohydrate metabolism in PCOS: a scoping review
Source: Front Endocrinol (Lausanne). 2026 Apr 13;17:1810805. doi: 10.3389/fendo.2026.1810805 (PMC13111087; doi:10.3389/fendo.2026.1810805)
Supplement: Supplementary file 3 [file SupplementaryFile1.docx]

Search queries for all databases

PubMed

("Polycystic Ovary Syndrome"[mh] OR pcos[tiab] OR "polycystic ovary syndrome"[tiab]) AND ( "Insulin Resistance"[mh] OR "Hyperinsulinism"[mh] OR "Glucose Metabolism Disorders"[mh] OR "Glycolysis"[mh] OR "Energy Metabolism"[mh] OR "glucose metabolism"[tiab] OR "carbohydrate metabolism"[tiab] OR "insulin resistance"[tiab] OR hyperinsulin*[tiab] OR "insulin signaling"[tiab] OR "insulin tolerance"[tiab] OR "glucose transporter*"[tiab] OR glut1[tiab] OR glut2[tiab] OR glut3[tiab] OR glut4[tiab] OR glut5[tiab] OR glut6[tiab] OR glut7[tiab] OR glut8[tiab] OR glut9[tiab] OR glut10[tiab] OR glut11[tiab] OR glut12[tiab] OR glut13[tiab] OR glut14[tiab] OR "glucose uptake"[tiab] OR glycolysis[tiab] OR "insulin sensitiv*"[tiab] OR "insulin secret*"[tiab] OR pi3k[tiab] OR akt[tiab] OR foxo*[tiab] OR "insulin receptor*"[tiab] OR irs[tiab] OR INSR*[tiab] OR "glucose production"[tiab] OR "beta cell*"[tiab] ) AND ( receptor*[tiab] OR pathway*[tiab] OR regulat*[tiab] OR upregulat*[tiab] OR downregulat*[tiab] OR crosstalk[tiab] OR interact*[tiab] OR mechanism*[tiab] OR "Signal Transduction"[mh] OR "Gene Expression"[mh] OR signal*[tiab] OR phosphor*[tiab] OR transcriptom*[tiab] OR proteom*[tiab] ) AND ("2018/01/01"[dp] : "2025/05/15"[dp])

N=1770

Embase

('polycystic ovary syndrome'/exp OR pcos:ti,ab,kw OR 'polycystic ovary syndrome':ti,ab,kw) AND ('insulin resistance'/exp OR 'hyperinsulinism'/exp OR 'glucose metabolism'/exp OR 'glucose metabolism disorder'/exp OR 'glycolysis'/exp OR 'energy metabolism'/exp OR 'glucose transport'/exp OR 'glucose metabolism':ti,ab,kw OR 'carbohydrate metabolism':ti,ab,kw OR 'insulin resistance':ti,ab,kw OR hyperinsulin*:ti,ab,kw OR 'insulin signaling':ti,ab,kw OR 'insulin tolerance':ti,ab,kw OR 'glucose transporter*':ti,ab,kw OR glut1:ti,ab,kw OR glut2:ti,ab,kw OR glut3:ti,ab,kw OR glut4:ti,ab,kw OR glut5:ti,ab,kw OR glut6:ti,ab,kw OR glut7:ti,ab,kw OR glut8:ti,ab,kw OR glut9:ti,ab,kw OR glut10:ti,ab,kw OR glut11:ti,ab,kw OR glut12:ti,ab,kw OR glut13:ti,ab,kw OR glut14:ti,ab,kw OR 'glucose uptake':ti,ab,kw OR glycolysis:ti,ab,kw OR 'insulin sensitiv*':ti,ab,kw OR 'insulin secret*':ti,ab,kw OR pi3k:ti,ab,kw OR akt:ti,ab,kw OR foxo*:ti,ab,kw OR 'insulin receptor*':ti,ab,kw OR irs*:ti,ab,kw OR INSR*:ti,ab,kw OR 'glucose production':ti,ab,kw OR 'beta cell*':ti,ab,kw) AND (receptor*:ti,ab,kw OR pathway*:ti,ab,kw OR regulat*:ti,ab,kw OR upregulat*:ti,ab,kw OR downregulat*:ti,ab,kw OR crosstalk:ti,ab,kw OR interact*:ti,ab,kw OR mechanism*:ti,ab,kw OR 'signal transduction'/exp OR 'protein phosphorylation'/exp OR 'gene expression'/exp OR signal*:ti,ab,kw OR phosphor*:ti,ab,kw OR transcriptom*:ti,ab,kw OR proteom*:ti,ab,kw) AND [01-01-2018 to 15-05-2025]/pd

N=3684

WOS

TS=( ("polycystic ovary syndrome" OR pcos) AND ( "insulin resistance" OR "hyperinsulinism" OR hyperinsulin* OR "glucose metabolism" OR "glucose metabolism disorder*" OR "glucose transport" OR "carbohydrate metabolism" OR glycolysis OR "energy metabolism" OR "insulin signaling" OR "insulin tolerance" OR "glucose transporter*" OR glut1 OR glut2 OR glut3 OR glut4 OR glut5 OR glut6 OR glut7 OR glut8 OR glut9 OR glut10 OR glut11 OR glut12 OR glut13 OR glut14 OR "glucose uptake" OR "insulin sensitiv*" OR "insulin secret*" OR pi3k OR akt OR foxo* OR "insulin receptor*” OR irs* OR INSR* OR "glucose production" OR "beta cell*" ) AND ( receptor* OR pathway* OR regulat* OR upregulat* OR downregulat* OR crosstalk OR interact* OR mechanism* OR "signal transduction" OR "protein phosphorylation" OR "gene expression" OR signal* OR phosphor* OR transcriptom* OR proteom* ) ) AND DOP=2018-01-01/2025-05-15

N=2607
